# Molecular Cloning, Functional Characterization, and Evolutionary Analysis of Vitamin D Receptors Isolated from Basal Vertebrates

**DOI:** 10.1371/journal.pone.0122853

**Published:** 2015-04-09

**Authors:** Erin M. Kollitz, Guozhu Zhang, Mary Beth Hawkins, G. Kerr Whitfield, David M. Reif, Seth W. Kullman

**Affiliations:** 1 Program in Environmental and Molecular Toxicology, Department of Biological Sciences, North Carolina State University, Raleigh, North Carolina, United States of America; 2 Bioinformatics Research Center, North Carolina State University, Raleigh, North Carolina, United States of America; 3 Department of Biological Sciences, North Carolina State University, Raleigh, North Carolina, United States of America; 4 Department of Basic Medical Sciences, University of Arizona College of Medicine, Phoenix, Arizona, United States of America; Laboratoire de Biologie du Développement de Villefranche-sur-Mer, FRANCE

## Abstract

The vertebrate genome is a result of two rapid and successive rounds of whole genome duplication, referred to as 1R and 2R. Furthermore, teleost fish have undergone a third whole genome duplication (3R) specific to their lineage, resulting in the retention of multiple gene paralogs. The more recent 3R event in teleosts provides a unique opportunity to gain insight into how genes evolve through specific evolutionary processes. In this study we compare molecular activities of vitamin D receptors (VDR) from basal species that diverged at key points in vertebrate evolution in order to infer derived and ancestral VDR functions of teleost paralogs. Species include the sea lamprey (*Petromyzon marinus*), a 1R jawless fish; the little skate (*Leucoraja erinacea*), a cartilaginous fish that diverged after the 2R event; and the Senegal bichir (*Polypterus senegalus*), a primitive 2R ray-finned fish. Saturation binding assays and gel mobility shift assays demonstrate high affinity ligand binding and classic DNA binding characteristics of VDR has been conserved across vertebrate evolution. Concentration response curves in transient transfection assays reveal EC_50_ values in the low nanomolar range, however maximum transactivational efficacy varies significantly between receptor orthologs. Protein-protein interactions were investigated using co-transfection, mammalian 2-hybrid assays, and mutations of coregulator activation domains. We then combined these results with our previous study of VDR paralogs from 3R teleosts into a bioinformatics analysis. Our results suggest that 1, 25D_3_ acts as a partial agonist in basal species. Furthermore, our bioinformatics analysis suggests that functional differences between VDR orthologs and paralogs are influenced by differential protein interactions with essential coregulator proteins. We speculate that we may be observing a change in the pharmacodynamics relationship between VDR and 1, 25D_3_ throughout vertebrate evolution that may have been driven by changes in protein-protein interactions between VDR and essential coregulators.

## Introduction

The ray-finned fishes (class: Actinopterygii) are the largest, most successful, and most diverse group of vertebrates [1]. Of the roughly 28,000 species in this class, fewer than 60 are not teleost fish. These few species comprise the four basal Actinopterygiian lineages of *Polypteriformes*, *Acipenseriformes*, *Lepisosteiformes*, and *Amiiformes*. These lineages are collectively referred to as “ancient fish” as they are considered to be living fossils. This is due to their morphology having remained unchanged over long periods of time and their lineages being relatively species poor [1, 2]. In contrast to their more ancient cousins, teleost fish display a far greater degree of morphologic variation and species diversity. It is likely that this diversity is a result of serial whole genome duplication (WGD) events that occurred early in their lineage. The serial “2R” genome duplication hypothesis suggests that the vertebrate genome is a result of two rapid and successive rounds of genome duplication (referred to as 1R and 2R) near the divergence of jawless and jawed vertebrates, approximately 500 million years ago [3]. Within ray-finned fishes, a third (3R) WGD occurred in the stem lineage of the teleostean fishes [2, 4]. WGDs are proposed to be a considerable force in vertebrate evolution by providing a source of raw genetic material for evolutionary forces to act upon, resulting in the functional divergence of new genes with novel functions [5]. The evolution and divergence of duplicate genes may result in larger gene families and permit more complex interactions and gene networks to evolve, leading to increased morphological complexity, adaptability, and speciation [5, 6]. This is consistent with the notion that larger genomes facilitate functional diversification and enable complex gene interactions. These processes in turn may facilitate morphological variation and physiological plasticity [7].

Duplicate genes have several possible fates. The classic model predicts that one copy is usually lost, due to functional redundancy and accumulated mutations as a result of relaxed selection. However, occasionally one copy will obtain a beneficial mutation that confers a novel function while the other copy maintains the original function, referred to as neofunctionalization [8]. A model hypothesized by Force et al, predicts that instead of one copy being lost, both copies undergo subfunctionalization [9]. Under this model, each copy may obtain complementary yet degenerative mutations, resulting in the partitioning of the ancestral function between the two copies, and the preservation of both copies to maintain the original function.

One class of regulatory genes that have demonstrated preferential retention post-WGD is the nuclear receptor superfamily [10–12]. Nuclear receptors (NRs) are ligand-activated transcription factors that regulate a diverse array of functions in metazoans; including metabolism, homeostasis, reproduction, embryonic development, and control of cellular proliferation and differentiation [13, 14]. NRs regulate gene expression through binding to small, lipophilic signaling molecules, resulting in systematic control and expression or silencing of target genes [14]. Such control facilitates cellular responses to exogenous and endogenous signals through the coordination of complex transcriptional processes.

Studies in teleost fish have identified orthologs for all mammalian NR subclasses, including those for steroid hormones receptors and orphan receptors [10–12, 15]. Furthermore, these studies have revealed that teleost fish maintain a larger complement of NRs than mammals. For example, 48 NRs have been identified in human [16], compared to 70 NRs identified in zebrafish (*Danio rerio*) [12], 71 NRs in the Japanese medaka (*Oryzias latipes*) [15], and 68 and 71 NRs in *Takifugu rubripes* and *Tetraodon nigroviridis*, two species of pufferfish [10, 11]. The greater number of NRs in teleosts is hypothesized to be due to global retention of NRs and other regulatory genes within the teleost genomes subsequent to the 3R event [5, 6, 17].

As the teleost-specific 3R event is in evolutionary terms more recent, these organisms are attractive models for the study of gene evolution and functional divergence post-WGD [18]. Previous studies of teleost NR paralogs have found evidence of functional divergence in fundamental NR functions, including ligand binding [19, 20], receptor transactivation [21–23], and protein-protein interactions [24]. Specifically, our group has identified evidence of functional divergence between teleost vitamin D receptor (VDR, NR1I1) paralogs with regards to protein-protein interactions between VDR paralogs and essential coregulator proteins, receptor transactivation in response to 1, 25D_3_, and DNA binding affinities [24]. However, the lack of data on ancestral VDRs limited our ability to speculate on ancestral vs. derived functions of the VDRα and VDRβ paralogs.

Prior to the identification of functional VDRs in aquatic vertebrates, the conventional theory speculated that the vitamin D endocrine system originated in terrestrial animals due to their complete dependency on dietary calcium [25]. However, studies suggest that the vitamin D endocrine system has an ancient origin. For example, functional VDRs have been cloned from the sea lamprey (*Petromyzon marinus*) [26], the little skate (*Leucoraja erinacea*) (this study) and numerous other fish [10, 11, 21, 27, 28]. Homology comparisons of vertebrate VDR protein sequences demonstrate that VDRs maintains a high degree of conservation across species, suggesting that the vitamin D endocrine axis may be highly conserved throughout vertebrate evolution [24, 26, 29]. Supporting this theory is the fact that many non-mammalian vertebrates maintain high concentrations of circulating vitamin D_3_, and enzymes necessary for vitamin D synthesis and transport have been identified in many species [30–32] (and further reviewed in [33, 34]). However, increasing evidence suggests that calcium homeostasis may not have been a critical function of ancestral VDR. The fact that aquatic vertebrates live in an environment that serves as an abundant calcium source, and functional VDRs have been identified in animals with and without calcified skeletons indicates that VDR may have evolved long before its role in hormonal control of calcium. Yet little is known regarding endocrine physiology of vitamin D in basal vertebrates.

In order to elucidate the evolutionary history and ancestral molecular functions of VDR, we have isolated and characterized VDRs from key lineages of basal vertebrates that diverged early in vertebrate evolution. For this study we have included two basal cartilaginous vertebrates: the sea lamprey (*Petromyzon marinus*), a 1R jawless fish (Agnatha) that is considered to be one of the most basal extant vertebrates [35], and the little skate (*Leucoraja erinacea*), a member of the class Chondrichthyes, which was the first lineage to diverge after the 2R duplication [36]. We have additionally included the Senegal bichir (*Polypterus senegalus*), a primitive ray-finned fish that diverged before the teleost 3R duplication, and thus maintains a single VDR ortholog [36, 37]. We hypothesize that comparison of VDR function across the deep ancestry of Agnatha, Chondrichthyes and basal Actinopterygii will help facilitate a conceptual framework of mechanisms underlying nuclear receptor innovation and comparative endocrine physiology.

## Materials and Methods

### DNA constructs

#### Little Skate VDR

Embryonic cDNA and an expressed sequence tag (EST) representing a complete open reading frame (ORF) of the VDR nucleic acid sequence from the little skate (*Leucoraja erinacea*) were gifts from Dr. Randall Dahn at Mount Desert Island Biological Laboratory (MDIBL, Salisbury Cove, ME). The skate VDR cDNA sequence was amplified using a nested PCR protocol. All PCR primers were designed using Primer3 software (S1 Table) [38]. The initial 50 μL PCR reaction included 1 μL skate embryonic cDNA, 1 μL each of forward and reverse outer primers (10 μM), 1 μL 50x dNTPs, 1 μL 50x Advantage Taq (Clontech Laboratories, Mountain View, CA), 5 μL 10x Advantage Taq buffer, and 41 μL PCR-grade water. The reaction was initially heated at 95°C for 2 minutes, followed by 40 cycles of 95°C for 30 seconds, 55.1°C for 30 seconds, 72°C for 1.5 minutes, and a final step of 72°C for 15 minutes. A single PCR product was excised from a 1.5% agarose gel and purified. An aliquot (1 μL) of the purified product was used as the DNA template for the second PCR reaction with the internal primer sets. The second reaction used identical thermocycle settings, however the annealing temperature was modified to match each internal primer set (59ºC for both pSG5 and pVP16, 63ºC for pET32a). PCR products were gel-purified and ligated into the pGEM-T easy vector (Promega Corporation, Madison, WI) according to the manufacturer’s recommendations. The GC10 strain of *Escherichia coli* bacteria (Genesee Scientific, San Diego, CA) were transformed with 50 ng of the ligation product, and positive clones were identified via a blue/white screen. A single colony was used to inoculate 3 mL LB/amp, and grown overnight at 37°C with shaking at 200 rpm. Plasmid DNA was isolated from the 3 mL cultures using the QIAGEN Hi-Speed miniprep kit (QIAGEN, Valencia, CA), and plasmids were sequenced to confirm gene identity and construct orientation. Full-length cDNA for skate VDR was further subcloned into the pSG5, pVP16, and pET32a vectors via restriction digest using incorporated restriction sites (see S1 Table), gel purified, and ligated into target vectors using T4 DNA ligase (Promega). All constructs were restriction mapped and sequenced to ensure identification, integrity, and orientation of each VDR within each expression vector. All constructs consist of the complete VDR ORF including internal start and stop codons, with the exception of pET32a. In this construct the internal stop codon was removed for inclusion of the 3’ polyhistidine-tag. The sequence data for skate VDR has been submitted to GenBank under the accession number KJ925051.

#### Senegal Bichir VDR

Senegal bichir (*Polypterus senegalus*) liver tissue was a provided by Dr. Kenneth Poss and Dr. Randall Dahn at MDIBL. This study was carried out in strict accordance with the recommendations in the National Academy of Sciences Guide for the Care and Use of Laboratory Animals. The protocol was approved by the Mount Desert Island Biological Laboratory Institutional Animal Care and Use Committee (Protocol Number: 10–12). Bichir were euthanized with tricaine methanesulfonate (300mg/L) in Phosphate Buffered Saline (PBS) (pH 7.0). All efforts were made to minimize suffering.

Liver tissue was homogenized in 1 mL of RNA Bee (Tele Test Inc, Friendswood, TX) using a Bullet Blender (Next Advance, Averill Park, NY). Following homogenization, total RNA was isolated following the manufacturer’s protocol. RNA quantity and 260/280 ratios were determined using a NanoDrop ND-1000 spectrophotometer (Thermo Fisher Scientific, Waltham, MA). First strand cDNA was synthesized using 2 μg total RNA and the High Capacity cDNA Reverse Transcription Kit (Life Technologies, Grand Island, NY) following the manufacturer’s instructions. PCR primers were designed using Primer3 [38] (S1 Table), and were based on an identified EST from the *Polypterus senegalus* sequence project headed by Dr. Ben King at MDIBL. Bichir VDR was amplified using Advantage Taq as described above, and each 50 μL reaction included 1 μL bichir cDNA, 1 μL each of forward and reverse primers (10 μM), 1 μL 50x dNTPs, 1 μL 50x Advantage Taq, 5 μL 10x Advantage Taq Buffer, and 41 μL PCR-grade water. Reactions were initially heated at 95°C for 2 minutes, followed by 30 cycles of 95°C for 30 seconds, 61.4°C for 30 seconds, 72°C for 2 minutes, and a final step of 72°C for 15 minutes. PCR products were resolved and excised from a 1.5% agarose gel, purified, and ligated into the pGEM-T Easy vector according to the manufacturer’s recommendations. The pGEM-bichir VDR construct was transformed into GC10 bacteria and purified as described above. Bichir VDR was further subcloned into pSG5, pVP16, and pET32a as described above. All constructs were restriction mapped and sequenced to ensure identification, integrity, and orientation of each VDR within each expression vector. The sequence data for bichir VDR has been submitted to GenBank under the accession number KJ925050.

#### Lamprey and Human VDR, Luciferase Reporters, and Coregulator Constructs

The pSG5-Lamprey VDR construct was a gift from Dr. Kerr Whitfield (University of Arizona, Phoenix, AZ). The pSG5-Human VDR construct was a gift from Dr. John Moore (GlaxoSmithKline, Research Triangle Park, NC). Full-length lamprey VDR and human VDR were further subcloned into the pVP16 and pET32a vectors as described above. All human coregulator transient transactivation and mammalian 2-hybrid constructs were a gift from Dr. Donald McDonnell (Duke University, Durham, NC). The XREM luciferase reporter, 5XGal4-TATA-Luc mammalian 2-hybrid luciferase reporter, and the pRL-CMV internal luciferase control were obtained as described previously [21, 24].

### Homology and Phylogeny

Vertebrate VDR sequences were obtained through BLAST analysis of the National Center for Biotechnology Information (NCBI; Bethesda, MD; www.ncbi.nih.gov/). Predicted full-length VDR amino acid sequences were identified, and aligned using ClustalW [39] via the SDSC Biology Workbench (URL: http://workbench.sdsc.edu). For the amino acid sequence alignment, VDR functional domains as well as key features of each domain were determined based on previous studies [40–42].

Phylogenetic analyses were conducted using MEGA 5.0 [43] and the maximum likelihood method based on the JTT model [44]. Trees were bootstrapped to assess robustness (500 pseudosamples). The percentage of replicate trees in which the associated taxa clustered together in the bootstrap test is shown next to the branches. The tree is drawn to scale, with branch length measured in the number of substitutions per site. The GenBank accession numbers for each VDR sequence can be found in S2 Table.

### Transient Transactivation Assays

To analyze transactivational activity of lamprey, skate, bichir, and human VDRs, full length VDR constructs were tested in transient transactivation assays with 1α, 25-dihydroxyvitamin D_3_ (1, 25D_3_, EMD Millipore, Billerica, MA) as the primary ligand. The assay protocol has been described in detail previously [24]. Briefly, HepG2 cells (ATCC #HB-8065) were seeded in 96-well plates (2.5 x 10^4^ cells/well) and transfected the following day using Lipofectamine 2000 (Life Technologies, Carlsbad, CA). Each well was transfected with 89.7 ng pSG5-VDR, 19.2 ng XREM-Luc reporter, and 4.5 ng pRL-CMV as an internal luciferase control. Coregulator studies included 18.3 ng of an expression vector containing the complete ORF of the human coregulator of interest (pCDNA-RXR_WT_ or RXR_AF2_, and/or pSG5-SRC1, GRIP1, or pCDNA-ACTR). Media was replaced 24 hours post-transfection with complete MEM spiked with 120 nM 1, 25D_3_ for single dose assays, or 0–1200 nM 1, 25D_3_ for concentration—response curves. Twenty-four hours post-exposure, the cells were passively lysed and luciferase activity was measured using the Dual-Glo Luciferase Assay System (Promega Corporation, Madison, WI) following the manufacturer’s protocol. Raw XREM-luciferase readings were first normalized to the pRL-CMV internal luciferase control. Following the initial normalization step, concentration-response data was normalized to the ethanol control. To enable a comparison of the presence and absence of coregulators on VDR transactivation, VDR response in the presence of coregulators was normalized to VDR response in the absence of any coregulators. Single-dose assays were analyzed using one-way ANOVAs followed by Tukey’s HSD post hoc test. Linear regression analysis using a sigmoidal dose-response calculation with variable slope was used to analyze concentration-response curves and determine median effective concentration (EC_50_), 95% confidence interval (95% CI), and the maximal efficacy (E_MAX_). All statistics were conducted in GraphPad Prism 4 (GraphPad Software, San Diego, CA). Experiments were repeated at least twice, and performed in replicates of four wells.

### Mammalian 2-Hybrid Assays

Protein-protein interactions between VDR and coregulator proteins were assessed using a mammalian 2-hybrid system (Clontech, Mountain View, CA), and has been described in detail previously [24]. Briefly, 96-well plates seeded with HepG2 cells were transfected with 33.6 ng pVP16-VDR as prey and 33.6 ng pM-coregulator as bait. The pM-coregulator is a fusion protein containing the yeast Gal4 DNA-binding domain fused to either full-length human RXR (pM-RXR_WT_ or pM-RXR_AF2_) or the defined NR box of the human SRC/p160 coactivators [45]: pM-SRC1_241-386_, pM-GRIP1_479-767_, or pM-ACTR_392-1005_. Each well additionally included 126.6 ng of the M2H luciferase reporter 5XGal4-TATA-Luc, which contains response elements for the yeast Gal4 DNA-binding domain, and 3 ng pRL-CMV as an internal luciferase control. Media was replaced 24 hours post transfection with complete MEM spiked with 120 nM 1, 25D_3_. Cells were tested for luciferase activity 24 hours post-exposure using the Dual-Glo Luciferase Assay System described previously. Raw 5xGal4-TATA-Luc readings were first normalized to the internal pRL-CMV control. Following the initial normalization, VDR-coregulator interaction was normalized to VDR in the absence of a coregulator construct. Results were analyzed via one-way ANOVAs followed by Tukey’s HSD post hoc test in GraphPad Prism 4 (GraphPad Software, San Diego, CA). Experiments were repeated at least twice and performed in replicates of four wells.

### Electrophoretic Mobility Shift Assays

Recombinant full-length VDR and RXR_WT_ proteins were expressed in a bacterial expression system and purified using immobilized metal affinity chromatography as described previously [24]. In brief, bacteria cultures were grown at 37°C/200 rpm until the OD_600_ reached 0.6. Protein expression was induced by the addition of 1 mM isopropyl-1-thio-β-galactopyranoside along with 20 μM ZnCl_2_ to increase protein solubility. Cultures were incubated for an additional 3 hours at 25°C/200 rpm. Following incubation, cultures were pelleted by centrifugation at 4,000 x g for 20 minutes at 4ºC. The supernatant was discarded, and the pellets were stored at -20°C until protein purification. Polyhistidine-tagged recombinant VDR and RXR_WT_ were purified under native kit conditions from the frozen pellet using the QIAexpress Ni-NTA Fast Start Kit (QIAGEN, Valencia, CA) according to the manufacturer’s protocol. Purified protein was quantified using a NanoDrop^®^ ND-1000 spectrophotometer (Thermo Fisher Scientific, Waltham, MA), and visualized via western blot using mouse monoclonal antibodies to the polyhistidine tags (#34660, QIAGEN, Valencia, CA).

DNA–protein binding reactions were performed as described previously [24]. Cy5-labeled and unlabeled oligos were purchased from Integrated DNA Technologies (Coralville, IA). The initial 20 μL binding reactions were composed of buffer (100 mM KCl, 10 mM HEPES, 1 mM EDTA, 0.1 mg/ml BSA, 4 μg/mL sonicated salmon sperm, 1.0 mM DTT, 1% glycerol, 20 mM MgCl_2_, pH 7.6), 100 ng recombinant VDR and 100 ng recombinant RXR_WT_ (in select assays), and 100 nM 1, 25D_3_ or ethanol as a vehicle control. Initial reactions were incubated for 45 minutes at 25°C. Following this incubation, 1 pmol of a 5’Cy5-labeled double-stranded oligo representing either the consensus sequence for the canonical vitamin D response element (VDRE) [46] (5’—AGCTTCAGGTCAAGGAGGTCAGAGAGC—3’) or a VDRE containing the DR3 found within the distal promoter region of the human CYP3A4-XREM reporter used in transient transfection studies (5’—GCTGAATGAACTTGCTGACCCTCTGCT—3’) [47] was added to each reaction. Competition reactions additionally included 100 pmol unlabeled wild-type XREM or canonical VDREs, or an unlabeled mutant form of the canonical sequence (5’—AGCTTCAG**AA**CAAGGAG**AA**CAGAGAGC—3’). Binding reactions were incubated for an additional 30 minutes at 25°C. Following incubation, 20 μL of each binding reaction was loaded into individual wells of a 6% native polyacrylamide gel, and resolved via nondenaturing gel electrophoresis for 90 minutes at 100 volts in 0.5x TBE buffer (45 mM Tris base, 45 mM boric acid, 1 mM EDTA, pH 8.0). Gels were visualized on a Storm 865 (GE Healthcare Life Sciences, Pittsburgh, PA). Negative controls include expressed affinity tags isolated from empty pET32a vector stocks, binding reactions run with either VDR or RXR individually, and ethanol as a vehicle control.

### Saturation Binding Analysis

Transfected cell lysates were prepared for saturation binding assays from Cos7 cells (ATCC #CRL-1561) as described in detail previously [24]. Cos7 cells (3.0 x 10^6^ cells per 150 mm culture dish) were transfected with 4 μg pSG5-VDR, 4 μg pSG5-RXR_WT_, and 16 μg of pBSII vector as carrier DNA, using Lipofectamine 2000. Media was replaced 24 hours post-transfection, and cells were harvested by trypsinization at 48 hours post-transfection. Trypsinized cells were washed twice in 2 mL ice-cold DPBS, and resuspended in 1 mL ice-cold KETZD + 5 buffer (0.15 M KCl, 1 mM EDTA, 10 mM Tris HCl, 0.3 mM ZnCl_2_, 200x dilution protease inhibitor cocktail (EMD Millipore, Billerica, MA), 5 mM DTT, pH 7.0). Resuspended cells were lysed by sonication (12 repetitions of 1-second bursts at 25% power), and centrifuged at 100,000 x g for 30 minutes at 4°C to pellet the cell debris. The supernatant containing the lysate was divided into aliquots and stored at -80°C.

The affinity of basal and human VDRs for 1, 25D_3_ was assessed as described previously [24]. Briefly, the lysate was diluted 1/20 in ice-cold KETZD+5 buffer, and 200 μL was transferred to each labeled reaction tube. Radiolabeled 1, 25-(OH)_2_-26, 27-[^3^H]dimethyl-vitamin D_3_ ([^3^H]-1, 25D_3_, original specific activity 157 Ci/mmol, Perkin Elmer, Waltham, MA) was diluted to 20 Ci/mmol with unlabeled 1 μM 1, 25D_3_, and further diluted with ethanol to obtain the desired concentrations (0–1.6 nM). The appropriate concentration of [^3^H]-1, 25D_3_ (10 μL) was added to each reaction, shaken, and incubated overnight at 4°C. Unbound ligand was removed with the addition of 80 μL of a 0.5% dextran-2.5% charcoal suspension in GP Buffer (150 mM NaCl, 15 mM NaN_3_, 100 mM anhydrous Na_2_HPO_4_, 39 mM NaH_2_PO_4_•H_2_O, 0.1% gelatin, pH 7.6) incubated in an ice bath for 15 minutes with brief shaking every 5 minutes. Samples were centrifuged at 5000 x g for 5 minutes at 4°C to pellet the charcoal suspension, and 200 μL of the supernatant containing the bound ligand was removed for liquid scintillation counting. Total binding was determined using lysate transfected with both pSG5-VDR and pSG5-RXR_WT_, and nonspecific binding was determined by using lysate transfected with the empty pSG5 vector in place of pSG5-VDR. Specific binding was calculated by subtracting the nonspecific binding counts from the total binding counts. Hyperbolic one-site binding curves were fit using GraphPad Prism 4. The reported dissociation constant (K_d_) for each species is the average ± SEM (n = 3 or 4). Variation between the average K_d_ values was tested via a one-way ANOVA followed by Tukey’s HSD post hoc test. All assays were repeated at least three times with duplicate tubes for each concentration.

### Bioinformatic summary analysis

In order to put these functional data in a broader context, the mammalian 2-hybrid (M2H) and transient transactivation (TT) data for lamprey, bichir, skate, medaka (VDRα and VDRβ), zebrafish (VDRα and VDRβ) and human VDR were integrated into a summary cluster analysis. The data for medaka and zebrafish VDRα and VDRβ are from our previous study [24]. The data for each assay were standardized as z-scores across all eight VDR species to account for interspecies differences in absolute assay readout. The entire data matrix, visualized as a two-color heatmap, was then subjected to unsupervised, hierarchical clustering using Manhattan distance and complete linkage. The Pickett Plot to the right of the heatmap indicates the presence/absence of co-regulators within each assay, as described earlier in Materials and Methods. Data were visualized using the R package Heatplus [48].

Next, to identify particular functional assays that were drivers of both the overall cluster pattern and species subclusters, a bootstrap permutation was implemented using custom R code [49]. For each of 10,000 bootstrap samples of assays, we counted the number of times the overall cluster pattern and subclusters (Lamprey, Bichir), (Medaka β, Skate, Zebrafish β), and (Zebrafish α, Medaka α, Human) were recapitulated. Across all permutations, assays having a large effect on cluster membership (e.g. permuting Assay_A_ significantly reduced the co-occurrence of species in Cluster_C_) were inferred to be important drivers of VDR functional similarity.

## Results

### Homology and Phylogeny

Here we report cloning of full-length VDR cDNA for both the little skate and the Senegal bichir. Skate VDR cDNA is composed of 1305 base pairs, encoding a predicted protein of 435 amino acids and a molecular weight of 49.4 kDa. Bichir VDR cDNA is composed of 1266 base pairs, encoding a predicted protein of 422 amino acids and a molecular weight of 47.9 kDa. Lamprey and human VDR have been cloned and described previously [26, 50]. Lamprey VDR is composed of 1221 base pairs that encode a predicted protein of 407 amino acids with a molecular weight of 46.0 kDa. Human VDR is composed of 1284 base pairs that encode a predicted protein of 428 amino acids with a molecular weight of 48.3 kDa.

**Fig 1:**
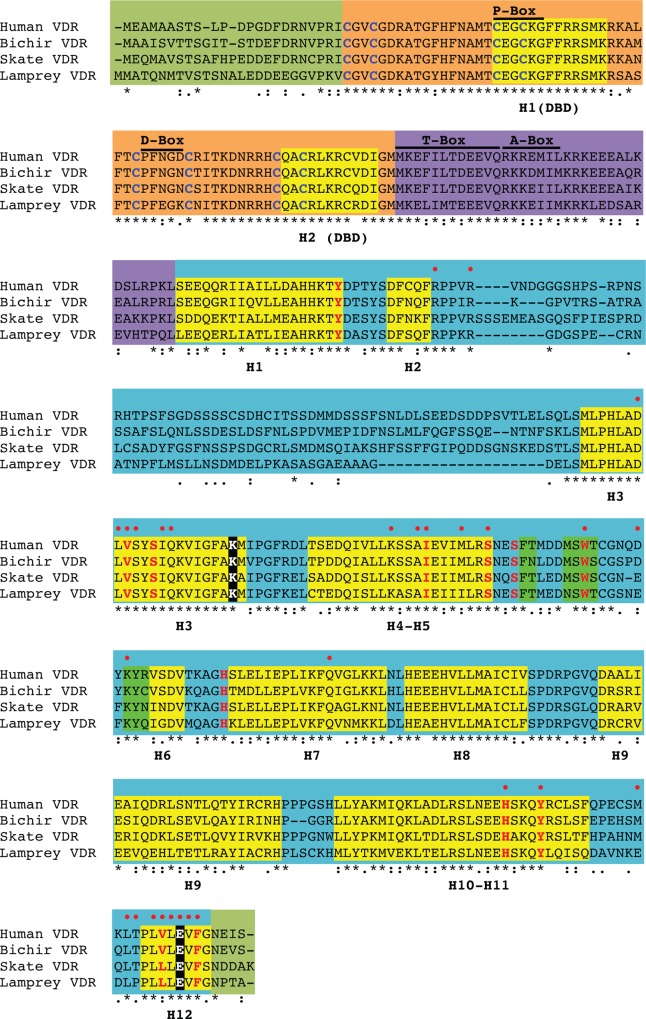
Amino acid sequence alignment of lamprey, skate, bichir, and human VDRs. Functional domains and domain elements were determined based on previous work with human VDR discussed in the manuscript. Fully conserved residues are indicated by “*”. Partial conservation is indicated by “:” (strong) or “.” (weak). Lack of residue consensus is indicated by a blank space. The five functional domains are shaded as follows: The N-terminal A/B domain and the C-terminal F domain are shaded in olive green. The DNA binding domain (DBD) is shaded in orange. The CTE/hinge domain is shaded in purple, while the ligand binding domain (LBD) is shaded in blue. For the DBD, residues that make up the P-box and D-box are indicated with a solid black line, and helix 1 (the recognition helix) and helix 2 are highlighted in yellow. Conserved cysteine residues involved in zinc finger structure are both bold and blue. For the CTE/hinge domain, the T-box and A-box are indicated with solid black lines. For the LBD, the estimated α-helices are highlighted in yellow and identified with the letter H followed by the helix number. The three β-sheets are highlighted in bright green. Amino acid residues that serve as contacts for 1, 25D_3_ are bold and red. The K and E residues in H3 and H12 involved in the charge clamp formation bold, white, and highlighted in black. A red circle above a residue indicates that the residue plays a role in direct interaction with other VDR residues upon ligand binding.

Alignments of predicted amino acid sequences demonstrated a high degree of similarity among lamprey, skate, bichir, and human VDR with other vertebrate VDRs (Tables 1–3 and Fig 1). The highest degree of similarity is found in the DNA binding domain (Table 1). The human VDR DBD shared an 87%, 90%, and 93% similarity with the DBDs of lamprey, skate, and bichir VDRs, indicating a high degree of conservation across species. The LBD is less well conserved, and a 60%, 62%, and 68% similarity is shared between human VDR and lamprey, skate, and bichir (Table 2). Full-length amino acid sequences of lamprey, skate, and bichir share a 62%, 66%, and 71% similarity with human VDR (Table 3). The basal VDRs are fairly similar to both teleost VDR paralogs, and do not appear to be more closely related to one paralog over the other. Fig 1 depicts an amino acid sequence alignment of the four VDRs with the different functional domains and key features highlighted.

**Table 1:** Homology of vertebrate VDR DNA-binding domains (DBDs).

	**H**	**R**	**C**	**A**	**F**	**Mβ**	**Mα**	**ZFβ**	**ZFα**	**B**	**ES**	**S**	**L**
**L**	87	87	87	87	87	89	92	90	90	89	87	87	100
**S**	90	90	90	90	90	90	92	92	93	93	96	100	
**ES**	90	90	90	90	90	90	92	92	93	93	100		
**B**	93	93	95	95	95	95	93	95	96	100			
**ZFα**	92	92	92	92	92	95	96	96	100				
**ZFβ**	92	92	92	92	92	96	96	100					
**Mα**	92	92	92	92	92	95	100						
**Mβ**	92	92	92	92	92	100							
**F**	95	95	95	95	100								
**A**	93	93	96	100									
**C**	96	96	100										
**R**	100	100											
**H**	100												

The numbers in the table correspond to percent sequence identity.

Key: Lamprey (L), Skate (S), Elephant Shark (ES), Bichir (B), Zebrafish α (ZFα), Zebrafish β (ZFβ), Medaka α (Mα), Medaka β (Mβ), Frog (F), Alligator (A), Chicken (C), Rat (R), Human (H).

**Table 2:** Homology of vertebrate VDR ligand-binding domains (LBDs).

	**H**	**R**	**C**	**A**	**F**	**Mβ**	**Mα**	**ZFβ**	**ZFα**	**B**	**ES**	**S**	**L**
**L**	60	59	59	59	57	60	61	61	57	59	56	59	100
**S**	62	61	62	62	64	62	64	63	60	62	66	100	
**ES**	62	63	62	64	60	65	64	65	61	62	100		
**B**	68	67	71	70	66	73	75	72	74	100			
**ZFα**	63	64	66	68	63	82	86	84	100				
**ZFβ**	66	67	66	69	64	85	85	100					
**Mα**	68	67	70	71	66	86	100						
**Mβ**	67	64	68	69	63	100							
**F**	68	68	73	79	100								
**A**	74	73	83	100									
**C**	72	74	100										
**R**	86	100											
**H**	100												

The numbers in the table correspond to percent sequence identity.

Key: Lamprey (L), Skate (S), Elephant Shark (ES), Bichir (B), Zebrafish α (ZFα), Zebrafish β (ZFβ), Medaka α (Mα), Medaka β (Mβ), Frog (F), Alligator (A), Chicken (C), Rat (R), Human (H).

**Table 3:** Homology of full-length vertebrate VDRs.

	**H**	**R**	**C**	**A**	**F**	**Mβ**	**Mα**	**ZFβ**	**ZFα**	**B**	**ES**	**S**	**L**
**L**	62	61	60	60	58	52	64	63	61	61	58	61	100
**S**	66	66	65	66	67	65	70	68	66	67	72	100	
**ES**	66	67	66	68	64	68	69	69	68	67	100		
**B**	71	71	75	74	70	76	77	74	76	100			
**ZFa**	68	68	70	71	66	83	86	84	100				
**ZFβ**	69	70	69	71	66	86	85	100					
**Mα**	71	72	73	74	69	85	100						
**Mβ**	70	69	70	70	66	100							
**F**	73	71	76	81	100								
**A**	79	78	86	100									
**C**	78	78	100										
**R**	90	100											
**H**	100												

The numbers in the table correspond to percent sequence identity.

Key: Lamprey (L), Skate (S), Elephant Shark (ES), Bichir (B), Zebrafish α (ZFα), Zebrafish β (ZFβ), Medaka α (Mα), Medaka β (Mβ), Frog (F), Alligator (A), Chicken (C), Rat (R), Human (H).

**Fig 2:**
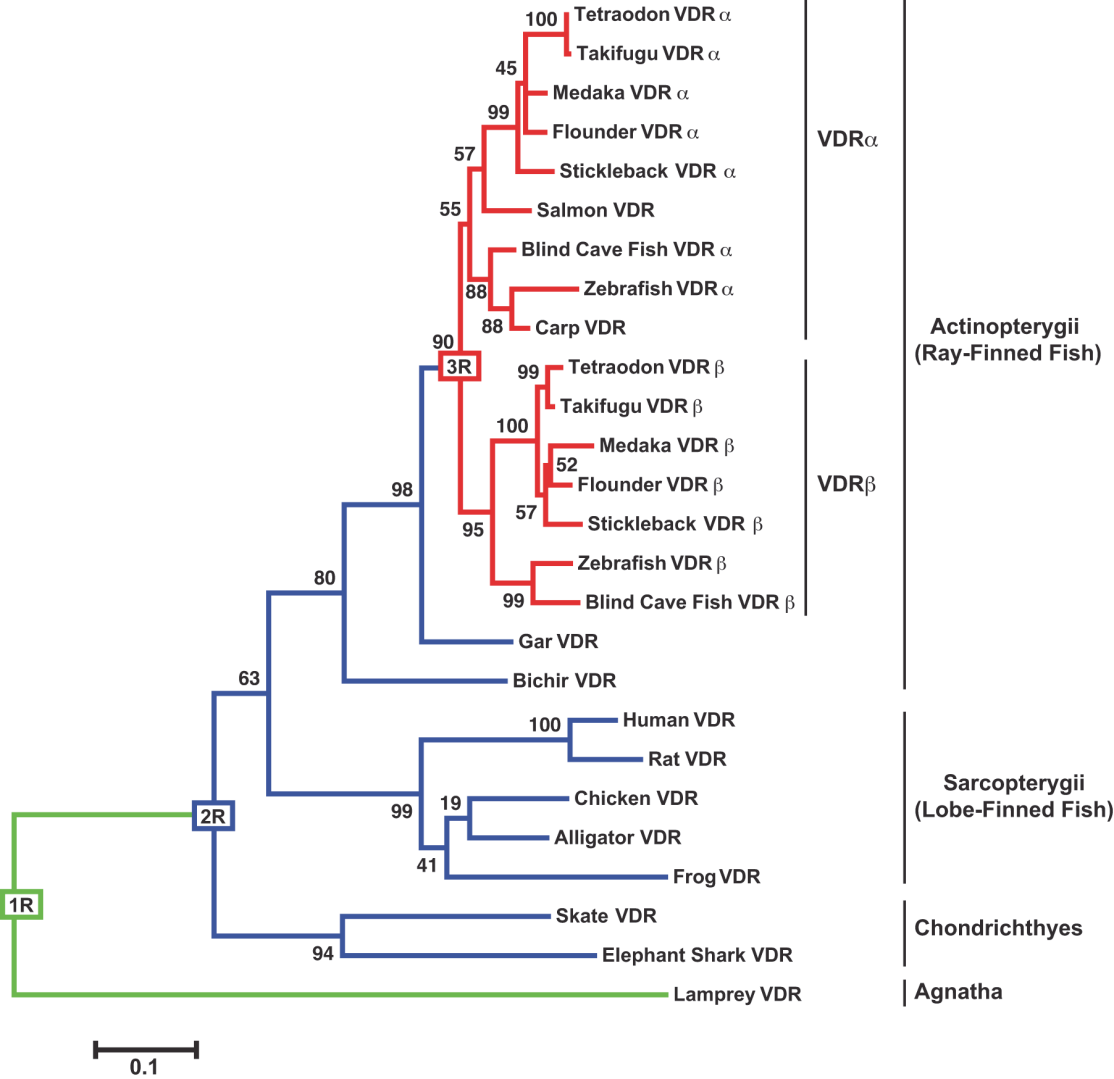
Phylogenetic analysis of full-length VDR amino acid sequences in vertebrates. Phylogenetic analysis was conducted in MEGA 5. This tree was estimated using the Maximum Likelihood method based on the JTT matrix-based model. The analysis was bootstrapped (500 pseudosamples) to assess robustness. The percentage of replicate trees in which the associated taxa cluster together in the bootstrap tests is illustrated at each node. The tree is drawn to scale, with branch lengths measured in the number of substitutions per site. The locations of each WGD (1R, 2R, and 3R) in vertebrate evolution are indicated on the tree and by branch color: green for 1R, blue for 2R, and red for 3R. The GenBank accession numbers for each VDR can be found in S2 Table.

Next, we conducted a phylogenetic analysis with full-length VDR sequences in order to compare phylogenetic relationships of the basal VDRs to additional VDRs throughout vertebrate evolution. VDR sequences include lamprey, skate and bichir, as well as medaka and zebrafish VDRα and VDRβ identified previously [24], and additional vertebrate VDR sequences identified in BLAST analysis. The organization of our tree is consistent with previously described hypothesis of vertebrate evolution (Fig 2) [1]. The species with calcified skeletons (Actinopterygii and Sarcopterygii) form a separate clade from the species with cartilaginous skeletons (Chondrichthyes and Agnatha). In addition, the Actinopterygii (ray-finned fish) and Sarcopterygii (lobe-finned fish, which includes tetrapods) formed two separate clades. Bichir VDR clustered with Actinopterygii, however both bichir and gar were excluded from the teleost cluster with a bootstrap probability of 80 and 98. VDRα and VDRβ form two separate subclusters within Actinopterygii, which is supported by bootstrap analysis (probability of 90) and is consistent with our previous report comparing the ligand-binding domains of teleost VDR paralogs and other tetrapods [21]. Human VDR clustered with the Sarcopterygii species and is closest to the other mammalian VDR (rat). Skate VDR significantly clustered with the other Chondrichthyes species (bootstrap probability of 94), and this cluster is basal to all other 2R species, which is consistent with Chondrichthyes representing the most basal lineage of jawed vertebrates. In addition, the chondrichthian VDR clade diverged before the Actinopterygii/Sarcopterygii split, suggesting that VDRs from skate and elephant shark are ancestral to both clades. Lamprey VDR appears to be the most divergent VDR based on branch length and homology comparisons, indicative of a greater number of amino acid changes in comparison to the other VDRs.

### **Transactivation of the basal VDRs in response to increasing concentrations of 1, 25D**_3_

**Fig 3:**
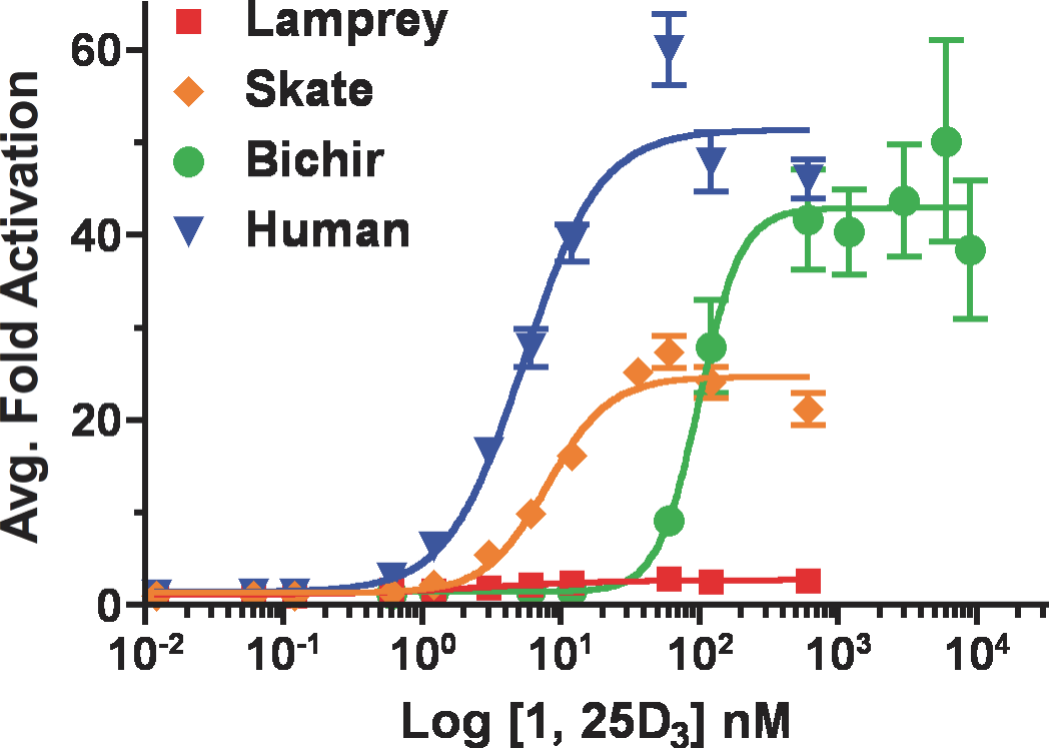
Transactivation of lamprey, skate, bichir, and human VDR in response to 1, 25D_3_. Concentration-response profiles are shown for lamprey VDR (red), skate VDR (orange), bichir VDR (green), and human VDR (blue). HepG2 cells were transiently transfected with full-length pSG5-VDR, the XREM-Luc reporter, and the pRL-CMV internal luciferase control as described previously in Materials and Methods. Cells were treated with 0–1200 nM 1, 25D_3_ in media for 24 hours. VDR response was measured via dual-luciferase assays. Data are represented as the mean fold activation normalized to the ethanol control ± SEM (n = 4). The half-maximal effective concentration (EC_50_), 95% confidence interval (95% CI), and maximum efficacy (E_MAX_) values for each VDR was determined using nonlinear regression analysis using a sigmoidal dose-response calculation with variable slope in Prism 4.

To analyze the differential transactivational activity of lamprey, skate, bichir, and human VDRs, full-length VDR constructs were tested in transient transactivation assays with increasing concentrations of 1, 25D_3_ (Fig 3). Observed EC_50_ values and 95% confidence intervals are as follows: 2.61 nM (1.42–4.79) for lamprey VDR, 7.93 nM (6.54–9.62 nM) for skate VDR, 98.92 nM (71.39–137.1 nM) for bichir VDR, and 5.38 nM (4.32–6.68 nM) for human VDR. Lamprey, skate, and human were all within the low nM range previously reported for both teleost VDR paralogs and human VDR [24, 29]. Conversely, EC_50_ value for bichir was much greater, at 98.9 nM. Significant variation was observed with the maximum efficacy (E_MAX_) values between VDRs (p < 0.0001, Fig 3). The E_MAX_ for lamprey was low (2.7 ± 0.1-fold). Skate exhibited an E_MAX_ of 24.6 ± 0.7-fold, whereas bichir and human VDR exhibited similar E_MAX_ values of 42.9 ± 2.2-fold and 51.3 ± 1.6-fold respectively.

### Ligand Binding Kinetics

**Fig 4:**
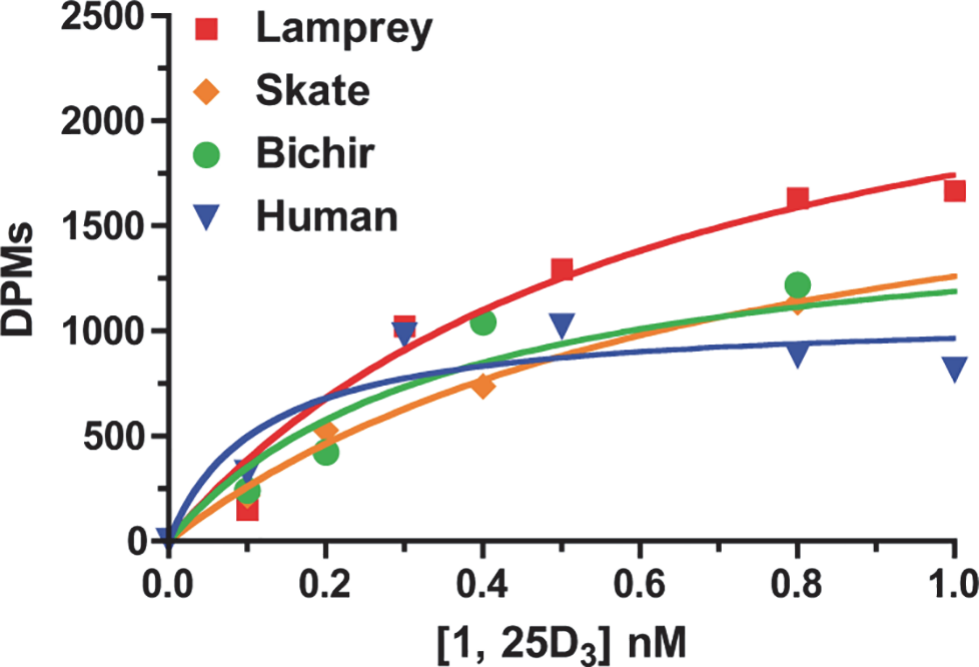
Saturation binding analysis of lamprey, skate, bichir, and human VDR to [^3^H]-1, 25D_3_. Saturation binding profiles are shown for lamprey VDR (red), skate VDR (orange), bichir VDR (green), and human VDR (blue). Lysates were prepared from transfected Cos7 cells as described in Materials and Methods. Lysates were incubated with 0–1.6 nM [^3^H]-1, 25D_3_ for 18 hours at 4°C. Unbound ligand was removed as described. Specific binding values were calculated by subtracting the average non-specific binding counts from the total binding counts. Hyperbolic one-site binding curves were fit using Prism 4. The reported dissociation constant (K_d_) in the manuscript for each VDR is the average of three or four separate experiments ± SEM. The graphs shown here are specific binding data from a representative experiment.

The affinity of the basal VDRs and human VDR was determined using saturation binding analysis with radiolabeled 1, 25D_3_ (Fig 4). Nonlinear regression analysis gave dissociation constant (K_d_) values of 0.47 ± 0.07 nM for lamprey (n = 4), 0.73 ± 0.10 nM for skate (n = 3), 0.40 ± 0.06 nM for bichir (n = 4), and 0.50 ± 0.07 nM for human VDR (n = 4). The K_d_ values obtained for each VDR were within the sub-nanomolar range previously reported for human and lamprey VDR, and teleost VDR paralogs [24, 26, 50]. No difference in binding affinities was detected between species (p>0.05), indicating a conservation of high affinity binding between VDR and 1, 25D_3_ across vertebrate evolution.

### DNA binding

**Fig 5:**
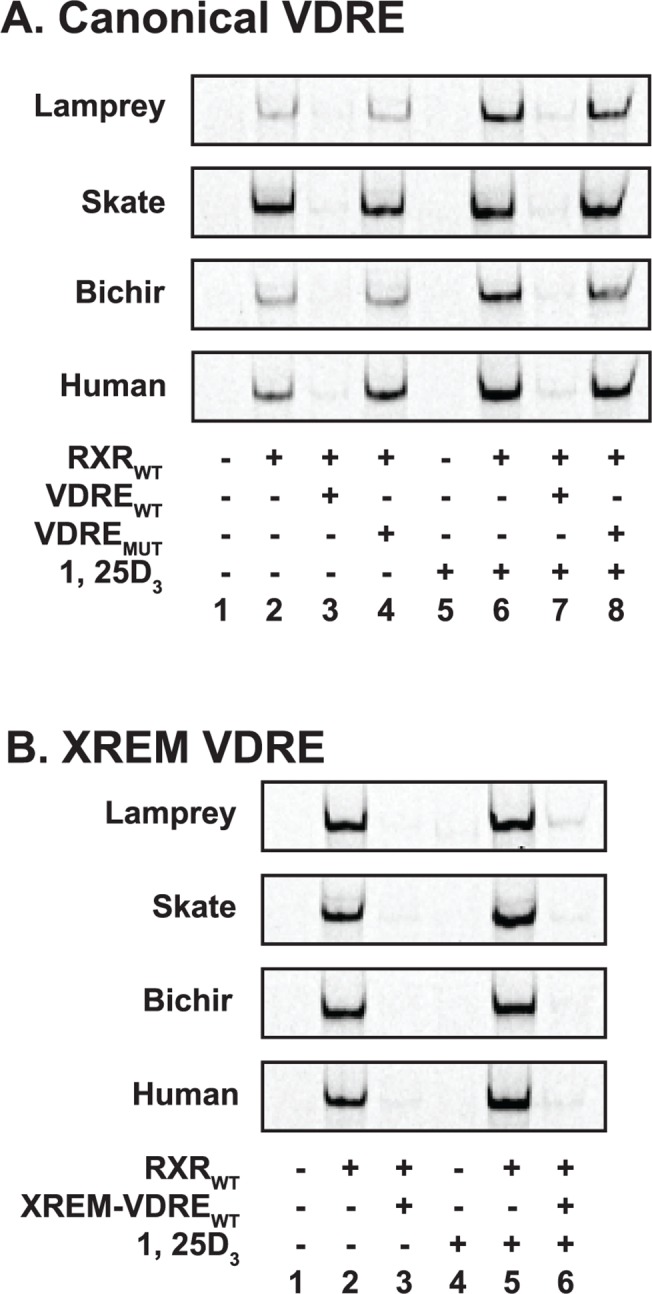
Electrophoretic mobility shift analysis of recombinant lamprey, skate, bichir, and human VDR. The above images depict VDR-RXR heterodimer binding to both (A) the canonical VDRE, and (B) the VDRE from the XREM region of CYP3A4. (A) With the canonical VDRE, DNA binding complexes were only observed in the presence of RXR_WT_ (lanes 2, 4, 6, 8 vs. lanes 1, 3, 5, 7). The addition of 100 nM 1, 25D_3_ (lanes 5–8) visibly enhanced binding complex formation compared to the ethanol control (lanes 1–4). In competition assays, the addition of 100-fold molar excess of the unlabeled wild type competitor (VDRE_WT_) successfully outcompeted binding to the labeled VDRE (lanes 2 vs. 3 and 6 vs. 7). The use of the mutant competitor (VDRE_MUT_) did not inhibit complex formation with any VDR tested (lanes 2 vs. 4 and 6 vs. 8). (B) A similar pattern was observed with the XREM VDRE. Complex formation was only observed in the presence of recombinant RXR_WT_ (lanes 1 vs. 2 and 4 vs. 5). The addition of 100 nM 1, 25D_3_ visibly enhanced complex formation (lane 2 vs. lane 5). In competition assays, the addition of 100-fold molar excess of the unlabeled XREM VDRE_WT_ successfully outcompeted complex formation on the labeled XREM VDRE (lanes 2 vs. 3 and 5 vs. 6).

To investigate VDR-RXR heterodimer association with DNA, electrophoretic mobility shift assays (EMSAs) were conducted with both canonical and non-canonical vitamin D response elements (VDREs) (Fig 5, uncropped images are provided in S1 File). With the canonical VDRE (Fig 5A), VDR binding was only observed in the presence of both VDR and RXR_WT_ (lanes 1 vs. 2, and 5 vs. 6). DNA binding did not occur when VDR or RXR_WT_ was used individually, indicating the necessity for obligate VDR-RXR_WT_ heterodimerization. Competition assays using wild-type unlabeled VDRE probes effectively outcompeted the VDR-RXR binding with labeled canonical VDRE, thus no binding complex was visible (lanes 2 vs. 3, and 6 vs. 7). Competition assays using an unlabeled mutant VDRE probe had no effect on VDR binding to the canonical VDRE (lanes 2 vs. 4 and 6 vs. 8). Addition of ligand (1, 25D_3_) visibly enhanced heterodimer binding to the canonical VDRE (lane 2 vs. lane 6).

To support transient transfection data, EMSAs were additionally conducted using a probe representing a divergent DR3 VDRE present within human CYP3A4 promoter, used in this study as the XREM-Luc reporter (Fig 5B). This VDRE has previously been demonstrated to facilitate expression of CYP3A4 and VDR transactivation of the XREM reporter [21]. As with the canonical DR3, all EMSAs with the XREM VDRE probe exhibited an obligate dependence on VDR-RXR heterodimerization facilitating VDR-DNA interactions (lanes 1 vs. 2, and 4 vs. 5). Competition assays using unlabeled probes effectively outcompeted VDR-RXR_WT_ binding with the XREM VDRE (lanes 2 vs. 3, and 5 vs. 6). Addition of 100 nM 1, 25D_3_ visibly enhanced heterodimer binding to the XREM VDRE tested in comparison to the ethanol control (lane 2 vs. 5).

### Basal VDR Heterodimerization with RXR

**Fig 6:**
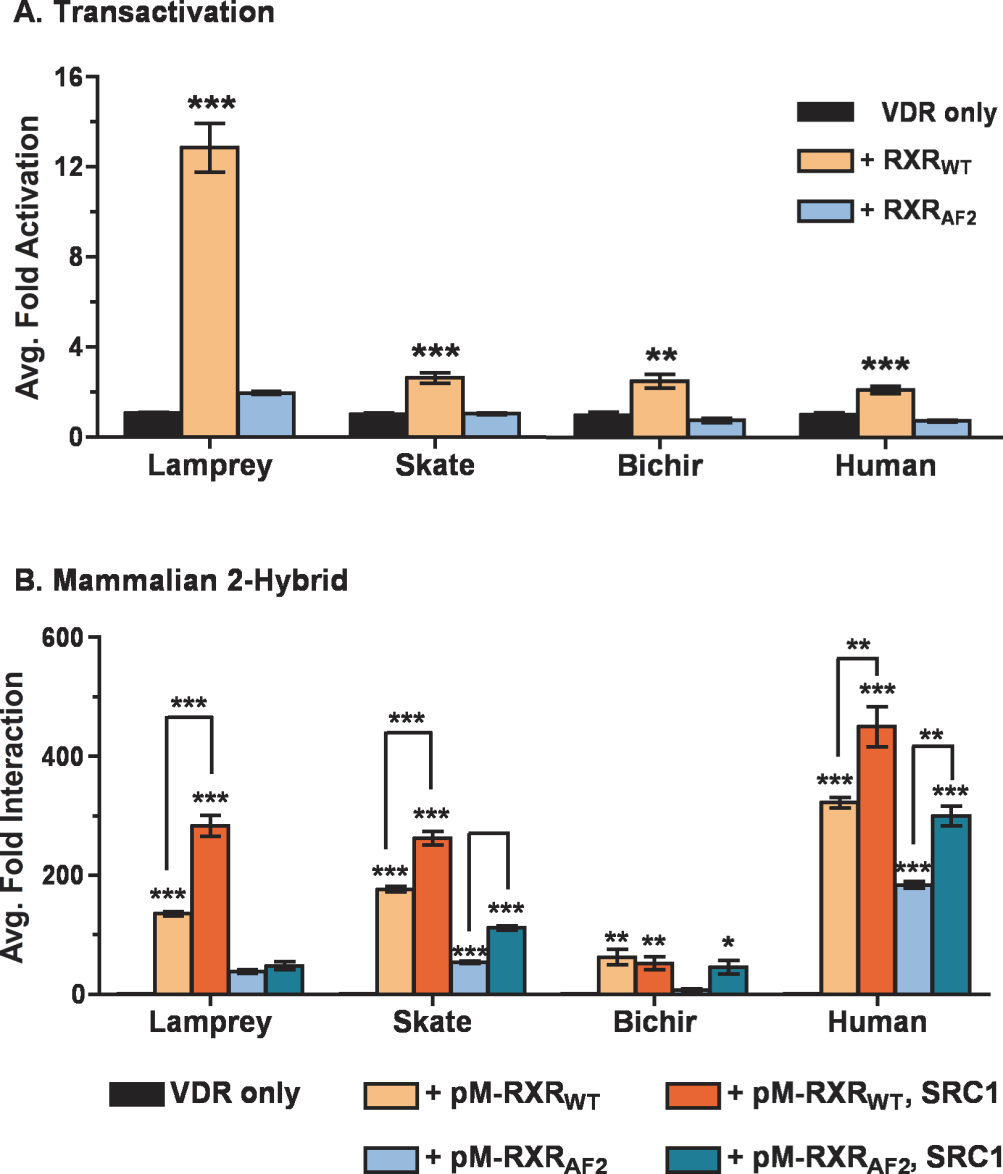
Assessment of VDR-RXR interactions. (A) Analysis of overexpressed RXR on VDR transactivation and (B) VDR-RXR heterodimerization in response to 1, 25D_3_. (A) HepG2 cells were transiently transfected with pSG5-VDR, the XREM-Luc reporter, and the pRL-CMV internal luciferase control as described previously in Materials and Methods. Select assays were cotransfected with pCDNA-RXR_WT_ (orange bars) or pCDNA-RXR_AF2_ mutant (blue bars). Cells were treated with 120 nM 1, 25D_3_ for 24 hours. VDR response was measured via dual-luciferase assays. Data are represented as the average fold activation normalized to VDR alone (no RXR) ± SEM (n = 4). Asterisks represent a significant increase in transactivation compared to the VDR control: *** = p < 0.001, ** = p < 0.01, * = p < 0.05. (B) Mammalian two-hybrid assays were conducted to study VDR-RXR heterodimerization in response to 1, 25D_3_. HepG2 cells were transiently transfected with pVP16-VDR as prey and pM-RXR_WT_ (light orange bars) or pM-RXR_AF2_ (light blue bars) as bait, along with the Gal4 luciferase reporter 5XGal4-TATA-Luc and the pRL-CMV internal luciferase control. Select experiments were also cotransfected with pSG5-SRC1 where indicated. Cells were exposed to 120 nM 1, 25D_3_ in media for 24 hours. Protein-protein interaction was measured via dual-luciferase assays as described in the Materials and Methods. Data are represented as the mean fold interaction ± SEM (n = 4). Data are normalized to VDR + empty pM vector (no RXR). Asterisks indicate significance: *** = p < 0.001, ** = p < 0.01, * = p < 0.05. Asterisks above brackets indicate the addition of pSG5-SRC1 significantly enhanced VDR-RXR interaction.

To assess the effects of RXR on VDR transactivation, additional transactivation studies were conducted utilizing VDR co-transfected with either full-length RXR_WT_ (pCDNA-RXR_WT_) or a truncated RXR mutant lacking the c-terminal AF2 region (pCDNA-RXR_AF2_). Results in Fig 6A illustrate that co-transfection with RXR_WT_ significantly increased VDR transactivation compared to VDR alone. Skate, bichir, and human VDR transactivation increased 2.1–2.6-fold over background, whereas lamprey VDR increased ~13-fold. In each instance where the RXR_AF2_ mutant was substituted for RXR_WT_, luciferase activity was significantly attenuated to background levels.

Next, mammalian 2-hybrid assays were conducted to determine if enhanced transactivation by RXR is due to direct protein-protein interactions (i.e. heterodimerization with VDR). As indicated in Fig 6B, pM-RXR_WT_ and pVP16-VDR demonstrated a strong and significant association in all species, indicating that each VDR is likely forming heterodimers with RXR_WT_ in response to 1, 25D_3_. The presence of pSG5-SRC1 significantly increased VDR-RXR association for lamprey, skate, and human VDR, but not bichir VDR. The use of the pM-RXR_AF2_ mutant significantly attenuated the association between pVP16-VDR and pM-RXR, although a weak but significant association was still demonstrated with skate, human, and bichir VDR in the presence of SRC1.

### Basal VDR Interaction with the SRC/p160 Family of Nuclear Receptor Coactivators

**Fig 7:**
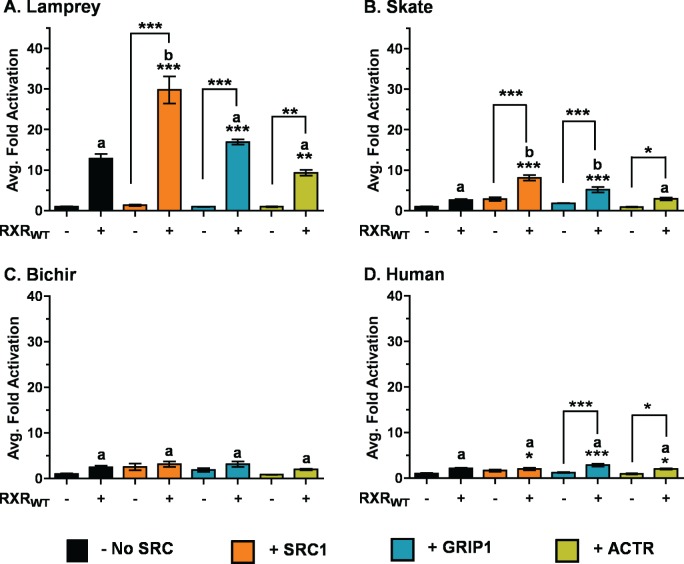
Influence of overexpressed SRC/p160 coactivators on VDR transactivation in response to 1, 25D_3_. The above graphs depict (A) lamprey VDR, (B) skate VDR, (C) bichir VDR, and (D) human VDR. HepG2 cells were transiently transfected with pSG5-VDR, the XREM-Luc reporter, and the pRL-CMV internal luciferase control as described previously in Materials and Methods. Select assays were cotransfected with pSG5-SRC1 (orange bars), pSG5-GRIP1 (blue bars), pSG5-ACTR (yellow bars), or no SRC/p160 coactivators (black bars). Assays were conducted both in the presence (+) and absence (-) of pCDNA-RXR_WT_. Cells were treated with 120 nM 1, 25D_3_ for 24 hours. VDR response was measured via dual-luciferase assays as described. Data are represented as the average fold activation normalized to VDR alone ± SEM (n = 4). Asterisks indicate significance: *** = p < 0.001, ** = p < 0.01, * = p < 0.05. Asterisks above brackets indicate the combination of RXR_WT_ the indicated SRC/p160 coactivator significantly increased VDR transactivation compared to the SRC/p160 coactivator in the absence of RXR_WT_. The letter “b” indicates the combination of both RXR_WT_ and the indicated SRC/p160 coactivator had a greater effect on VDR transactivation compared with VDR + RXR_WT_ in the absence of the SRC/p160 coactivator (black bar). The letter “a” indicates that no difference in VDR transactivation was observed in the presence of both RXR_WT_ and the indicated SRC/p160 coactivator vs. VDR + RXR_WT_ in the absence of the SRC/p160 coactivator. (p < 0.05).

Given that nuclear receptor coactivators are critical to VDR function, we next sought to investigate if co-transfection of VDR with members of the SRC/p160 family of nuclear receptor co-activators could facilitate VDR transactivation. In the absence of cotransfected RXR_WT_, the addition of SRC1, GRIP1, or ACTR did not affect VDR transactivation for any VDR tested (Fig 7A–7D). Conversely, the combination of RXR_WT_ and SRC1 resulted in a significant increase in VDR transactivation for lamprey and skate that was greater then the transactivation increase observed with either coregulator used individually (Fig 7A and 7B). A similar increase was observed with the cotransfection of RXR_WT_ and GRIP1 with skate VDR, but not lamprey. A significant increase was observed with human VDR co-transfected with RXR_WT_ and SRC1, GRIP1, and ACTR, but these increases were not significant when compared to VDR + RXR_WT_ in the absence of SRC/p160 coactivators (Fig 7D). Cotransfection with the SRC/p160 coactivators both in the presence and absence of RXR_WT_ did not affect bichir VDR transactivation with any of the coactivator combinations tested (Fig 7C).

**Fig 8:**
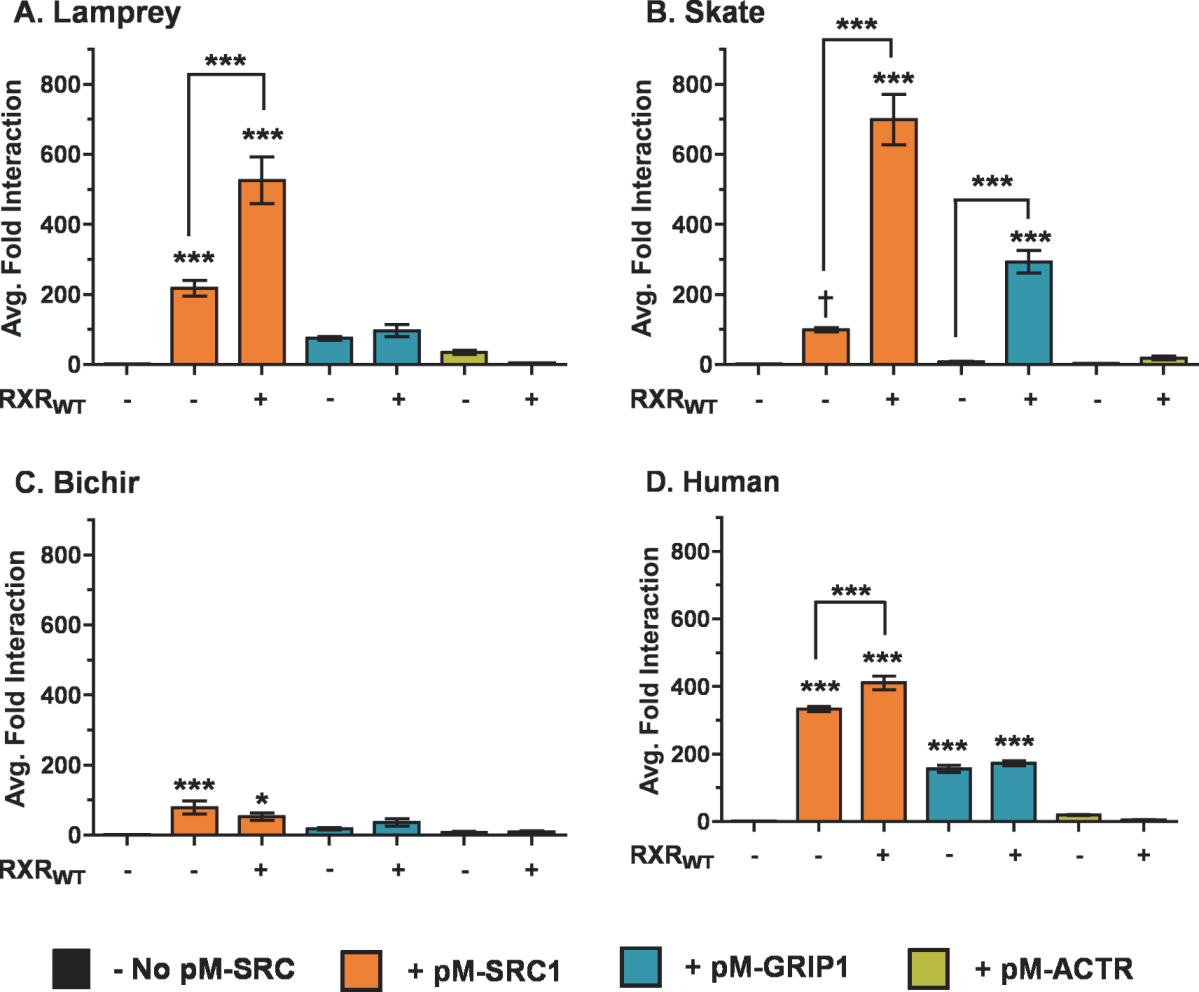
Mammalian 2-hybrid analysis of VDR interaction with SRC/p160 coactivators in response to 1, 25D_3_. The above graphs depict (A) lamprey VDR, (B) skate VDR, (C) bichir VDR, and (D) human VDR. Assays were conducted both in the presence (+) and absence (-) of pCDNA-RXR_WT_. HepG2 cells were transiently transfected with pVP16-VDR as prey and pM-SRC1 (orange bars), pM-GRIP1 (blue bars), or pM-ACTR (yellow bars) as bait, along with the 5XGal4-TATA-Luc reporter and pRL-CMV as an internal luciferase control. Cells were exposed to 120 nM 1, 25D_3_ in media for 24 hours. Protein-protein interaction was measured via dual-luciferase assays as described in the Materials and Methods. Data are represented as the mean fold interaction ± SEM (n = 4). Data are normalized to VDR + empty pM vector (no coactivators). Asterisks above bars represent a significant interaction between VDR and the SRC/p160 coactivators: *** = p < 0.001, ** = p < 0.01, * = p < 0.05. Asterisks above brackets indicate the addition of RXR_WT_ significantly enhanced VDR-SRC/p160 interaction. The interaction between pVP16-skate VDR and pM-SRC1 was tested with an unpaired t-test: t_6_ = 16.56, p < 0.0001 (†).

As with RXR, mammalian 2-hybrid assays were conducted to test the ability of the VDRs to directly recruit the SRC/p160 coactivators in response to 1, 25D_3_. Studies were conducted both in the presence and absence of supplemented RXR_WT_. Results from this study demonstrate that all VDRs are capable of forming direct interactions with pM-SRC1 (Fig 8A–8D). Further, interactions between lamprey, skate, and human VDR and pM-SRC1 and were significantly enhanced in the presence of co-transfected full-length RXR_WT_. Human was the only VDR tested that demonstrated direct interaction with pM-GRIP1. Conversely, cotransfection with RXR_WT_ resulted in a significant association between GRIP1 and skate VDR. None of the VDRs demonstrated interaction with pM-ACTR in the presence of absence of RXR_WT_.

### Summary of Bioinformatic Analysis

**Fig 9:**
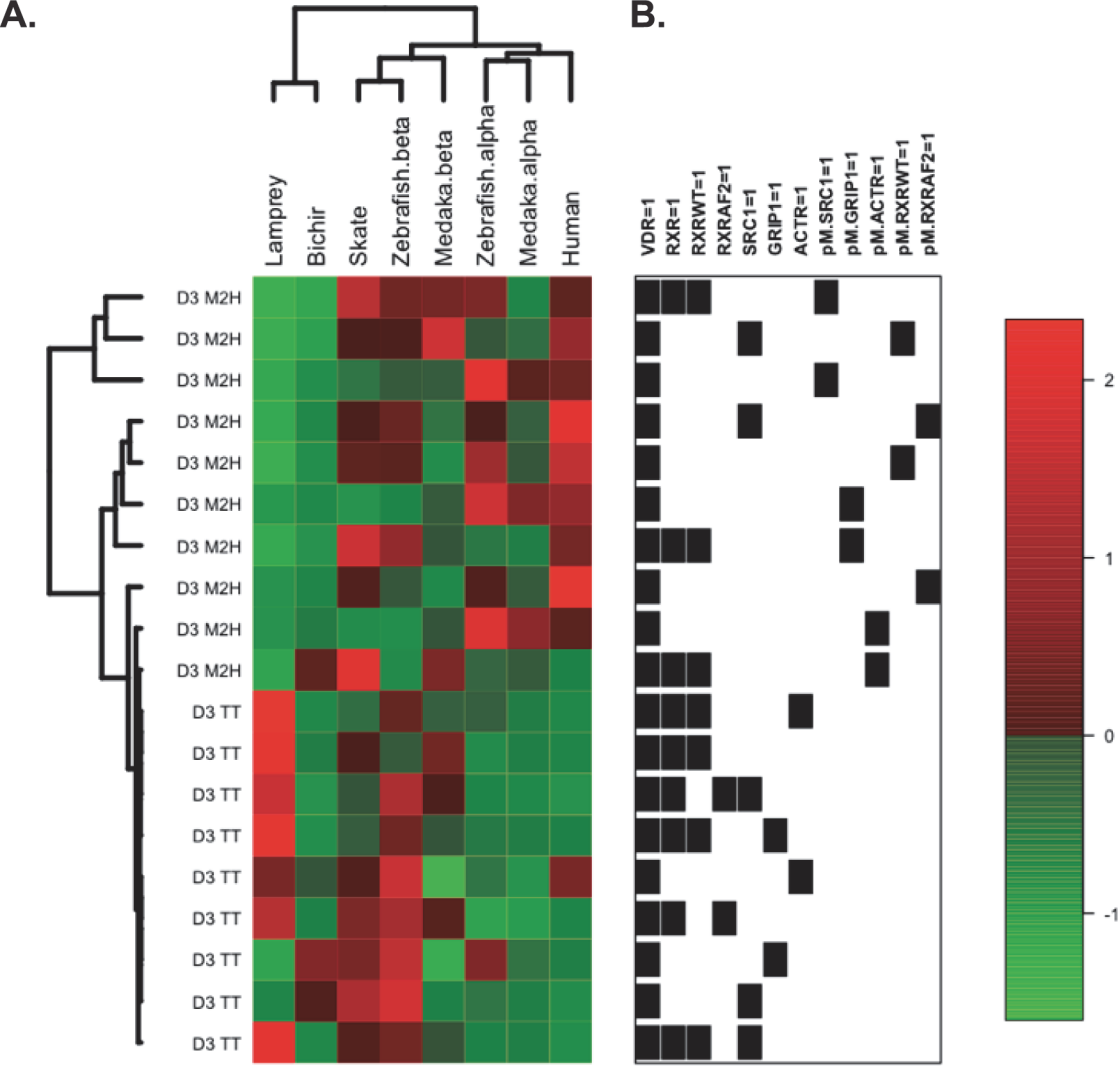
Hierarchical clustering of transient transactivation (TT) and mammalian 2-hybrid (M2H) assays. (A) Clustering includes eight VDR orthologs with Manhattan distance and complete linkage. Data for each assay (rows) were standardized as z-scores across all eight VDR species (columns) to account for interspecies differences in absolute assay readout. (B) The Pickett Plot to the right of the heatmap indicates the presence/absence of coregulators within each assay.

Fig 9A and 9B provides a global, multispecies context for VDR functional assays that includes previous data for medaka and zebrafish VDRα and VDRβ [24]. The data resulted in three empirical clusters of [Lamprey, Bichir], [Skate, Zebrafish β, and Medaka β] and [Zebrafish α Medaka α, and Human]. The first cluster (C1) included Lamprey and Bichir, which shared similar response across most of the 1, 25D_3_ mammalian 2-hybrid assays (M2H). However, the two species differ in their activity in the transient transfection (TT) assays. The next cluster (C2) included Skate and the VDRβ paralogs of Zebrafish and Medaka. The last cluster (C3) grouped Humans with the VDRα paralogs of Zebrafish and Medaka.

Permutation stability results were used to infer factors underlying the empirical clustering results. Activity associated with the coregulators GRIP1, SRC1, and ACTR drove assignment of the two Medaka paralogs (VDRα and VDRβ) to separate clusters. When assays involving these factors were permuted, only ~ 20% of resulting clusters maintained intact C3 membership. Cluster C2 was less sensitive to permutation, although SRC1 and GRIP1 cofactors disrupted the inclusion of Zebrafish VDRβ in this cluster. In this analysis, Lamprey and Bichir clustered together in 99% of permutations, regardless of functional assay(s) involved.

## Discussion

Nuclear receptors (NRs) are ligand-dependent transcription factors that bind to lipophilic signaling molecules, resulting in systematic control and expression of target genes. Such control facilitates cellular responses to endogenous and exogenous signals through the coordination of complex transcriptional processes [51]. Despite their diversity in function, NRs share a common protein organization including a highly conserved N-terminal DNA binding domain (DBD) and a C-terminal ligand-binding domain (LBD), and specific transactivation domains [14] (Fig 1). The identification of NRs in both diploblastic Cnidarian and triploblastic animals suggests NR proteins arose early in metazoan evolution [52]. This is supported by the fact that NRs appear to be absent in genomes of fungi, plants, and prokaryotes, and suggests that NRs arose in multicellular organisms sometime after the divergence of metazoans from lower eukaryotes [53]. Phylogenetic comparisons of ancestral NRs demonstrate that during evolution to bilaterian lineages, NR diversification was shaped by two major periods of gene loss and duplication and lineage-specific expansion [52]. The first period lead to the divergence of the six major subfamilies. The second period occurred specifically in vertebrates, and resulted in paralogs within each subfamily. The second period of diversification is consistent with the vertebrate serial genome duplication hypothesis, which states that the vertebrate genome is a result of successive rounds of genome duplication [54].

While significant information is available regarding functionality of most mammalian NRs, there is a paucity of data regarding NR functions of basal vertebrates. This data gap limits our ability to infer derived and ancestral properties of more recent receptor functions. We have previously reported evidence of functional divergence between vitamin D receptor paralogs (VDRα and VDRβ) isolated from teleost fish that evolved after the 3R WGD event [24]. In order to elucidate the evolutionary history and ancestral molecular functions of VDR, we have characterized VDR function from basal vertebrates that diverged at key periods in vertebrate evolution. Our results indicate that select characteristics of VDR function, such as receptor-ligand interactions, evolved early and have been highly conserved throughout vertebrate evolution. However, VDR transactivation in response to 1, 25D_3_, and the influence of protein-protein interactions between nuclear receptor coactivators and VDR appear to vary significantly between basal and more derived vertebrates.

We first confirmed that all three basal VDRs and human VDR are capable of 1, 25D_3_-dependent transactivation by titrating the concentration of 1, 25D_3_ in our transactivation assays. Combined with our previous data on medaka and zebrafish VDRα and VDRβ [24], EC_50_ values for all VDRs (with the exception of bichir) were in the low nanomolar range (<10 nM). These results are consistent with that observed from other VDR studies which employed Gal4-VDR-LBD chimeras to assess VDR transactivation across phylogenetically diverse taxa [21, 29]. The EC_50_ for bichir, a 2R bony fish, was over 10-fold greater than the EC_50_ for any other VDR tested, suggesting that 1, 25D_3_ is a less potent agonist for this VDR ortholog. The reason for the decreased potency observed in bichir VDR is unclear. The fact that the EC_50_ for VDRs from species that diverged both before and after bichir are in the low nanomolar range suggests that the decreased sensitivity observed with bichir VDR might be species or lineage specific. However, attenuated sensitivity of *Xenopus laevis* VDR to 1, 25D_3_ has also been previously reported [25]. While 1, 25D_3_ is a highly potent VDR ligand for the vast majority of species tested, the results with bichir and *Xenopus* indicate that potency may vary between species. Mechanistic details associated with differential ligand sensitivities are currently undetermined, yet VDR of both species retain 1, 25D_3_-mediated transactivation, RXR heterodimerization, and DNA binding [25].

Our concentration-response curves further suggest that the ability of 1, 25D_3_ to induce a highly efficacious response is likely a more recent evolutionary innovation. High transactivational efficacy was only observed in the bony vertebrates (i.e. bichir, human, and VDRα), compared to lamprey and skate (and VDRβ), which appears to be more ancestral-like in function. The maximum transactivational efficacy was highest for human and bichir, which is similar to the efficacies observed for medaka and zebrafish VDRα from our previous study [24]. The maximum transactivational efficacy was much lower for lamprey and skate, the two most basal vertebrate species. Our lamprey transactivation data is consistent with the previous findings reported by Whitfield et al [26], and Reschly et al [29], which demonstrated that lamprey VDR maintains an EC_50_ comparable to that of human and rodent VDR, however the maximum level of transactivation exhibited by lamprey VDR was highly attenuated by comparison.

The observed differential transactivational efficacies are potentially associated with critical NR functions including ligand binding, DNA binding, receptor heterodimerization and recruitment of coregulators to the transcription complex. The perturbation of any of these steps may have critical effects on downstream VDR transactivation. We thus ran additional assays to assess each of these fundamental NR functions with VDR orthologs from basal vertebrates. We first conducted saturation binding assays with basal VDRs and [^3^H]-1, 25D_3_ to determine if the observed differential transactivational efficacies were potentially a result of differential ligand affinities. Decreased ligand affinity may be indicative of a ligand’s ability to stabilize VDR in the active conformation, which may have downstream effects on subsequent steps in receptor transactivation. Yet ligand-binding data reveal that ligand affinity between 1, 25D_3_ and VDR is well conserved across vertebrate evolution, including basal species. In each instance, K_d_ values for all VDRs tested were within the sub-nanomolar range (10^–10^ M) previously reported for high affinity mammalian VDR proteins [55]. Of note, lamprey and skate had slightly lower ligand affinities compared to bichir and human. The lower affinity of lamprey and skate VDR is consistent with previously reported ligand affinity by Whitfield et al [26] and may be associated with a leucine to valine substitution in both species compared to human VDR at Val418 [40]. The interaction between Val418 in human and 1, 25D_3_ stabilizes helix 12 (H12) of VDR in the active conformation, positioning it for optimal interaction with NR coactivators such as SRC1 [40]. The positioning of H12 is critical for NR activation, a necessity illustrated by the fact that destabilization of H12 has been determined to be a mechanism of action for some VDR antagonists [56], and that the loss of the AF2 region of H12 completely abolishes VDR transactivation [57]. However, in this study we have demonstrated that both lamprey and skate VDR are both highly capable of recruiting coactivators in response to 1, 25D_3_, and thus we suspect that such a small difference in ligand affinity is not driving the observed transactivational variances. Furthermore, it should be noted that ligand affinity is not necessarily indicative of transactivational efficacy. It has been previously demonstrated both by our laboratory and others that high ligand affinity and high transactivational efficacy of specific NR ligands are not proportional [22, 24, 58]. This phenomenon is likely due to the ability of ligands to induce subtle conformational differences within receptors that elicit different biological responses such as DNA binding, coactivator recruitment and heterodimerization [58].

RXR is considered the obligate heterodimer partner for VDR, and previous studies have demonstrated the necessity of this partnership in VDR-mediated transcription [59, 60]. The cotransfection of RXR_WT_ in our VDR transactivation assays significantly increased VDR activation for all VDRs tested. This is consistent with our previous study on teleost VDR paralog transactivation with RXR, and with enhanced VDR transactivation previously reported with human VDR in the presence of RXR [24, 61]. Our mammalian 2-hybrid assays additionally demonstrate that all VDRs directly heterodimerize with RXR_WT_ in response to 1, 25D_3_. When compared to our previous data with medaka and zebrafish VDRα and VDRβ[24] it appears the overexpression of RXR_WT_ increases VDR activation 2.1–5.4 fold in our system, similar to an ~1.6 fold increase previously observed in transactivation studies with cotransfected human VDR and RXR [61]. However, lamprey VDR proved to be exceptionally sensitive to RXR_WT_. Lamprey VDR transactivation increased ~13-fold in the presence of overexpressed RXR_WT_. These results are in contrast to a previous lamprey VDR study [26]. This study observed a much smaller increase in lamprey VDR transactivation with zebrafish RXR, and a lack of heterodimer formation with human RXR in DNA binding assays, although it should be noted that the lack of heterodimer formation may be due to the different VDRE sequences used in the previous study [26]. Our EMSA results provide additional support regarding the conservation and necessity of the VDR-RXR partnership for DNA binding. Analogous to previous observations with human VDR [60, 61], all basal vertebrate VDRs only formed binding complexes in the presence of RXR_WT_, and complex binding to VDREs was enhanced with the addition of 1, 25D_3_, emphasizing the necessity of ligand binding for optimal heterodimerization. Considering that all VDRs demonstrate increased transactivation in the presence of RXR, heterodimerize with RXR in response to 1, 25D_3_, and bind to both canonical and divergence VDREs with a high degree of specificity implies that VDR-RXR heterodimerization is an ancient trait that evolved early in VDR evolution, and has been highly conserved across species.

In addition to RXR, assays were run to determine the ability of the basal VDRs to recruit members of the SRC/p160 family of NR coactivators, and the effect of the coactivators on VDR transactivation. Leucine-rich motifs of the SRC/p160 coactivators, referred to as the NR box, interact with the AF2 region of NRs in a ligand-dependent manor [62]. SRC/p160 coactivators enhance NR transactivation through chromatin remodeling and recruiting additional proteins to the transcription complex [45]. Similar to our studies with RXR, our transactivation and mammalian 2-hybrid assays with the SRC/p160 coactivators also demonstrate a strong degree of conservation. All VDRs demonstrate a preference for SRC1 over GRIP1 and ACTR, a preference that has been observed previously with human VDR [63]. In our system, overexpressed RXR_WT_ was necessary for the SRC/p160 coactivators to enhance transactivation. Consistent with Jurutka et al [63], we have hypothesized that a direct association between these proteins is necessary to drive gene expression. Conversely, SRC/p160 coactivators did not have a significant effect on bichir VDR activation, although our M2H data indicate that bichir successfully recruits SRC1.

The ability of 1, 25D_3_ to bind the basal VDRs with high affinity yet only induce partial efficacy in transactivation assays compared to modern vertebrate VDRs suggests that 1, 25D_3_ may function as a partial agonist for the basal VDRs (and possibly the teleost VDRβ paralogs). Partial agonists are defined by their ability to directly bind a receptor, but are only able to induce partial efficacy compared to a full agonist. While these observations are traditionally made with a single receptor and multiple ligands, what we may be observing is a change in the pharmacodynamics relationship between VDR and 1, 25D_3_ throughout vertebrate evolution. Our lamprey and skate VDR data supports the theory that the evolution of 1, 25D_3_ as a high potency and high affinity ligand for VDR evolved early, however it appears that the ability of 1, 25D_3_ to act as a full agonist evolved later in time. Our bioinformatic analysis suggests that interaction with RXR and the SRC/p160 coactivators may have been influential in the ability of VDR to mediate a full agonist response to 1, 25D_3_. While VDR has maintained a high ligand affinity across species, we speculate that an increasing sensitivity to coregulators may have been an influence driving the increased transactivational response observed in both human and the teleost VDRα paralogs. The cluster analysis indicates that the coregulators have the greatest effect on the C3 cluster (human VDR, zebrafish and medaka VDRα). By examining phylogeny of our test species there appears to be a trend suggesting that full transcriptional response to 1,25D_3_ may have appeared before the Actinopterygii/Sarcopterygii split, but after the divergence of more basal jawed vertebrates such as Chondrichthyes, but additional studies are necessary to support this theory.

It is well known that vitamin D is an ancient hormone which is found in all forms of life [34]. That being said, the ancestral physiological role(s) for VDR and the vitamin D endocrine system in aquatic vertebrates remains relatively unknown. For bony fish, a canonical role for VDR and vitamin D in calcium ion homeostasis and skeletal maintenance may be inferred and for some teleost species initial evidence for this role has been demonstrated [27]. Yet in cartilaginous vertebrates, the vitamin D endocrine system likely has little role in skeletal maintenance, as these species lack mineralized bone. Rather, there is a proposed role for VDR in bile salt metabolism, detoxification, and immune system function in these species [26]. It is well established in terrestrial organisms that the vitamin D endocrine system extends far beyond skeletal maintenance. Studies over the past decade have revealed that VDR and the vitamin D endocrine system encompass many non-calcemic roles, including detoxification, embryonic development, cell proliferation and differentiation, neurodevelopment and immune system function (reviewed in [64, 65]). We speculate that some of these same roles for vitamin D and VDR may be functional in early aquatic vertebrates. These functionalities may even predate the role of vitamin D in calcium ion homeostasis and skeletal maintenance. In fact, a recent study by Lin et al [27] demonstrated that the VDRα paralog from zebrafish facilitates calcium uptake, bone mineralization and expression of vitamin D catabolizing CYPs, while the VDRβ paralog maintains a non-calcemic role [27]. This physiological data may in turn support our functional assessments demonstrating a definitive cluster between medaka and zebrafish VDRα and human VDR, which maintain calcemic modalities. Comparatively, medaka VDRβ, zebrafish VDRβ and skate VDR form a cluster that may represent a group of non-calcemic receptors. This diversification of teleosts VDR represents either neofunctionalization [8] in this case a role in calcium ion regulation, or subfunctionalization [9] depending upon the physiological role(s) of VDR in basal and ancestral vertebrates, which is yet to be determined [24]. Here we demonstrate that VDR evolved an increasing sensitivity to interactions with coregulator proteins during the evolution of bony vertebrates. We propose that this process may have in turn been a major influence driving the increased efficacy of VDR in response to 1, 25D_3_ observed in modern vertebrates and facilitated novel calcemic vitamin D endocrine functions compared to VDRs from ancestral species.

## Supporting Information

S1 Table List of primers. (PDF)

S2 Table GenBank Accession Numbers. (PDF)

S1 File Uncropped images of EMSA gels. (PDF)

## References

[1] Nelson JS. Fishes of the World. 4th Edition ed. New York: J. Wiley; 2006.

[2] Hoegg S, Brinkmann H, Taylor J, Meyer A. Phylogenetic timing of the fish-specific genome duplication correlates with the diversification of teleost fish. J Mol Evol. 2004;59(2):190–203. 10.1007/s00239-004-2613-z PubMed PMID: 000223424800004. 15486693

[3] Dehal P, Boore J. Two rounds of whole genome duplication in the ancestral vertebrate. Plos Biol. 2005;3(10):1700–8. 10.1371/journal.pbio.0030314 PubMed PMID: 000232404600005. PMC1197285 16128622

[4] Crow K, Stadler P, Lynch V, Amemiya C, Wagner G. The "fish-specific" Hox cluster duplication is coincident with the origin of teleosts. Mol Biol Evol. 2006;23(1):121–36. 10.1093/molbev/msj020 PubMed PMID: 000233843900015. 16162861

[5] Taylor J, Raes J. Duplication and divergence: The evolution of new genes and old ideas. Annu Rev Genet. 2004;38:615–43. PubMed PMID: 000226244600019. 15568988 10.1146/annurev.genet.38.072902.092831

[6] Crow K, Wagner G. What is the role of genome duplication in the evolution of complexity and diversity? Mol Biol Evol. 2006;23(5):887–92. 10.1093/molbev/msj083 PubMed PMID: 000237320800007. 16368775

[7] Van de Peer Y, Maere S, Meyer A. OPINION The evolutionary significance of ancient genome duplications. Nat Rev Genet. 2009;10(10):725–32. 10.1038/nrg2600 PubMed PMID: 000269965100015. 19652647

[8] Ohno S. Evolution by gene duplication New York: Springer-Verlag; 1970.

[9] Force A, Lynch M, Pickett F, Amores A, Yan Y, Postlethwait J. Preservation of duplicate genes by complementary, degenerative mutations. Genetics. 1999;151(4):1531–45. PubMed PMID: 000079560100024. 10101175 10.1093/genetics/151.4.1531 PMC1460548

[10] Maglich J, Caravella J, Lambert M, Willson T, Moore J, Ramamurthy L. The first completed genome sequence from a teleost fish (Fugu rubripes) adds significant diversity to the nuclear receptor superfamily. Nucleic Acids Res. 2003;31(14):4051–8. 10.1093/nar/gkg444 PubMed PMID: 000184106700029. 12853622 PMC165959

[11] Metpally RPR, Vigneshwar R, Sowdhamini R. Genome inventory and analysis of nuclear hormone receptors in Tetraodon nigroviridis. J Biosciences. 2007;32(1):43–50. PubMed PMID: 000243858400005. 10.1007/s12038-007-0005-4 17426379

[12] Bertrand S, Thisse B, Tavares R, Sachs L, Chaumot A, Bardet PL, et al Unexpected novel relational links uncovered by extensive developmental profiling of nuclear receptor expression. PLoS genetics. 2007;3(11):e188 Epub 2007/11/14. 10.1371/journal.pgen.0030188 17997606 PMC2065881

[13] Gronemeyer H, Gustafsson J, Laudet V. Principles for modulation of the nuclear receptor superfamily. Nat Rev Drug Discov. 2004;3(11):950–64. 10.1038/nrd1551 PubMed PMID: 000224833100019. 15520817

[14] Aranda A, Pascual A. Nuclear hormone receptors and gene expression. Physiol Rev. 2001;81(3):1269–304. Epub 2001/06/28. . 11427696 10.1152/physrev.2001.81.3.1269

[15] Howarth DL. Characterization of FXR alpha in medaka and its involvement in hepatobiliary injury [Dissertation] Durham: Duke University; 2009.

[16] Robinson-Rechavi M, Carpentier AS, Duffraisse M, Laudet V. How many nuclear hormone receptors are there in the human genome? Trends in genetics: TIG. 2001;17(10):554–6. Epub 2001/10/05. . 11585645 10.1016/s0168-9525(01)02417-9

[17] Edger PP, Pires JC. Gene and genome duplications: the impact of dosage-sensitivity on the fate of nuclear genes. Chromosome Res. 2009;17(5):699–717. 10.1007/s10577-009-9055-9 PubMed PMID: 000270448800010. 19802709

[18] Schartl M. Beyond the zebrafish: diverse fish species for modeling human disease. Disease Models and Mechanisms. 2014;7(2):181–92. 10.1242/dmm.012245 24271780 PMC3917239

[19] Hawkins MB, Thomas P. The unusual binding properties of the third distinct teleost estrogen receptor subtype ERbetaa are accompanied by highly conserved amino acid changes in the ligand binding domain. Endocrinology. 2004;145(6):2968–77. Epub 2004/03/06. 10.1210/en.2003-0806 . 15001543

[20] Jones BB, Ohno CK, Allenby G, Boffa MB, Levin AA, Grippo JF, et al New Retinoid-X Receptor Subtypes in Zebra Fish (Danio-Rerio) Differentially Modulate Transcription and Do Not Bind 9-Cis Retinoic Acid. Molecular and Cellular Biology. 1995;15(10):5226–34. PubMed PMID: ISI:A1995RV77200003. 7565671 10.1128/mcb.15.10.5226 PMC230770

[21] Howarth DL, Law SHW, Barnes B, Hall JM, Hinton DE, Moore L, et al Paralogous vitamin D receptors in teleosts: Transition of nuclear receptor function. Endocrinology. 2008;149(5):2411–22. 10.1210/en.2007-1256 PubMed PMID: 000255200000041. 18258682 PMC2329287

[22] Bury N, Sturm A, Le Rouzic P, Lethimonier C, Ducouret B, Guiguen Y, et al Evidence for two distinct functional glucocorticoid receptors in teleost fish. J Mol Endocrinol. 2003;31(1):141–56. PubMed PMID: 000185203800013. 12914532 10.1677/jme.0.0310141

[23] Ogino Y, Katoh H, Kuraku S, Yamada G. Evolutionary History and Functional Characterization of Androgen Receptor Genes in Jawed Vertebrates. Endocrinology. 2009;150(12):5415–27. 10.1210/En.2009-0523 PubMed PMID: ISI:000272042300026. 19819965 PMC2795718

[24] Kollitz EM, Hawkins MB, Whitfield GK, Kullman SW. Functional diversification of vitamin d receptor paralogs in teleost fish after a whole genome duplication event. Endocrinology. 2014;155(12):4641–54. Epub 2014/10/04. 10.1210/en.2014-1505 . 25279795 PMC4239418

[25] Li YC. Cloning and Characterization of the Vitamin D Receptor from Xenopus laevis. Endocrinology. 1997;138(6):2347–53. 10.1210/en.138.6.2347 9165021

[26] Whitfield G, Dang H, Schluter S, Bernstein R, Bunag T, Manzon L, et al Cloning of a functional vitamin D receptor from the lamprey (Petromyzon marinus), an ancient vertebrate lacking a calcified skeleton and teeth. Endocrinology. 2003;144(6):2704–16. 10.1210/en.2002-221101 PubMed PMID: 000183146900066. 12746335

[27] Lin CH, Su CH, Tseng DY, Ding FC, Hwang PP. Action of vitamin D and the receptor, VDRa, in calcium handling in zebrafish (Danio rerio). Plos One. 2012;7(9):e45650 Epub 2012/10/03. 10.1371/journal.pone.0045650 23029160 PMC3446910

[28] Suzuki T, Suzuki N, Srivastava A, Kurokawa T. Identification of cDNAs encoding two subtypes of vitamin D receptor in flounder, Paralichthys olivaceus. Biochem Bioph Res Co. 2000;270(1):40–5. PubMed PMID: 000086348900007. 10.1006/bbrc.2000.2378 10733902

[29] Reschly EJ, Bainy ACD, Mattos JJ, Hagey LR, Bahary N, Mada SR, et al Functional evolution of the vitamin D and pregnane X receptors. BMC Evol Biol. 2007;7:222 10.1186/1471-2148-7-222 . 17997857 PMC2263054

[30] Kobayashi T, Takeuchi A, Okano T. An evolutionary aspect in vertebrates from the viewpoint of vitamin D3 metabolism In: Norman AW, Bouillon R, Thomasset M, editors. Vitamin D: Gene Regulation, Structure-Function Analysis and Clinical Application. Berlin: Walter de Gruyter; 1991.

[31] Takeuchi A, Kobayashi T. Vitamin D3 in cartilaginous fish: identification, quantification, and metabolism of vitamin D3 In: Norman AW, Bouillon R, Thomasset M, editors. Vitamin D: Gene Regulation, Structure-Function Analysis and Clinical Application. Berlin: Walter de Gruyter; 1991.

[32] Pierens SL, Fraser DR. The origin and metabolism of vitamin D in rainbow trout. The Journal of steroid biochemistry and molecular biology. 2015;145:58–64. Epub 2014/10/12. 10.1016/j.jsbmb.2014.10.005 . 25305412

[33] Lock E-J, Waagbo R, Bonga SW, Flik G. The significance of vitamin D for fish: a review. Aquacult Nutr. 2010;16(1):100–16. 10.1111/j.1365-2095.2009.00722.x PubMed PMID: 000273449600011.

[34] Bouillon R, Suda T. Vitamin D: calcium and bone homeostasis during evolution. Bonekey Rep. 2014;3:480 Epub 2014/01/28. 10.1038/bonekey.2013.214 24466411 PMC3899559

[35] Escriva H, Manzon L, Youson J, Laudet V. Analysis of lamprey and hagfish genes reveals a complex history of gene duplications during early vertebrate evolution. Molecular biology and evolution. 2002;19(9):1440–50. Epub 2002/08/30. . 12200472 10.1093/oxfordjournals.molbev.a004207

[36] Kikugawa K, Katoh K, Kuraku S, Sakurai H, Ishida O, Iwabe N, et al Basal jawed vertebrate phylogeny inferred from multiple nuclear DNA-coded genes. BMC Biol. 2004;2:3 Epub 2004/04/09. 10.1186/1741-7007-2-3 15070407 PMC387836

[37] Inoue JG, Miya M, Tsukamoto K, Nishida M. Basal actinopterygian relationships: a mitogenomic perspective on the phylogeny of the "ancient fish". Mol Phylogenet Evol. 2003;26(1):110–20. Epub 2002/12/10. . 12470943 10.1016/s1055-7903(02)00331-7

[38] Rozen S, Skaletsky H. Primer3 on the WWW for general users and for biologist programmers In: Krawetz S, Misener S, editors. Bioinformatics Methods and Protocols: Methods in Molecular Biology. Totowa, NJ: Humana Press; 2000. 10.1385/1-59259-192-2:365 10547847

[39] Thompson JD, Higgins DG, Gibson TJ. CLUSTAL W: improving the sensitivity of progressive multiple sequence alignment through sequence weighting, position-specific gap penalties and weight matrix choice. Nucleic Acids Res. 1994;22:4673–80. 7984417 10.1093/nar/22.22.4673 PMC308517

[40] Rochel N, Wurtz J, Mitschler A, Klaholz B, Moras D. The crystal structure of the nuclear receptor for vitamin D bound to its natural ligand. Mol Cell. 2000;5(1):173–9. PubMed PMID: 000085163400016. 10678179 10.1016/s1097-2765(00)80413-x

[41] Adachi R, Shulman A, Yamamoto K, Shimomura I, Yamada S, Mangelsdorf D, et al Structural determinants for vitamin D receptor response to endocrine and xenobiotic signals. Mol Endocrinol. 2004;18(1):43–52. 10.1210/me.2003-0244 PubMed PMID: 000187624600004. 14525957

[42] Shaffer PL, Gewirth DT. Structural analysis of RXR-VDR interactions on DR3 DNA. J Steroid Biochem Mol Biol. 2004;89-90(1–5):215–9. 10.1016/j.jsbmb.2004.03.084 . 15225774

[43] Tamura K, Peterson D, Peterson N, Stecher G, Nei M, Kumar S. MEGA5: Molecular Evolutionary Genetics Analysis using Maximum Likelihood, Evolutionary Distance, and Maximum Parsimony Methods. Molecular Biology and Evolution. 2011;28:2731–9. 10.1093/molbev/msr121 21546353 PMC3203626

[44] Jones DT, Taylor WR, Thornton JM. The rapid generation of mutation data matrices from protein sequences. Computer Applications in the Biosciences. 1992;9:275–82. 10.1093/bioinformatics/8.3.275 1633570

[45] Leo C, Chen J. The SRC family of nuclear receptor coactivators. Gene. 2000;245(1):1–11. PubMed PMID: 000085869700001. 10713439 10.1016/s0378-1119(00)00024-x

[46] Shaffer P, Gewirth D. Vitamin D receptor-DNA interactions. Vitam Horm. 2004;68:257–73. PubMed PMID: 000223083600009. 15193458 10.1016/S0083-6729(04)68009-5

[47] Drocourt L, Pascussi J, Assenat E, Fabre J, Maurel P, Vilarem M. Calcium channel modulators of the dihydropyridine family are human pregnane X receptor activators and inducers of CYP3A, CYP2B, and CYP2C in human hepatocytes. Drug Metab Dispos. 2001;29(10):1325–31. PubMed PMID: 000171227200011. 11560876

[48] Ploner A. Heatplus: Heatmaps with Row and/or Column Covariates and Colored Clusters. R package version 260. 2012.

[49] RCoreTeam. R: A Language and Environment for Statistical Computing. Vienna, Austria: R Foundation for Statistical Computing; 2013 10.3758/s13428-013-0330-5

[50] Baker AR, McDonnell DP, Hughes M, Crisp TM, Mangelsdorf DJ, Haussler MR, et al Cloning and expression of full-length cDNA encoding human vitamin D receptor. Proceedings of the National Academy of Sciences of the United States of America. 1988;85(10):3294–8. Epub 1988/05/01. 2835767 10.1073/pnas.85.10.3294 PMC280195

[51] Laudet V, Gronemeyer H. The Nuclear Receptor Factsbook. San Diego: Academic Press; 2002.

[52] Escriva H, Bertrand S, Laudet V. The evolution of the nuclear receptor superfamily. Essays Biochem. 2004;40:11–26. PubMed PMID: ISI:000223268600002. 15242336 10.1042/bse0400011

[53] Bridgham JT, Eick GN, Larroux C, Deshpande K, Harms MJ, Gauthier ME, et al Protein evolution by molecular tinkering: diversification of the nuclear receptor superfamily from a ligand-dependent ancestor. PLoS biology. 2010;8(10). Epub 2010/10/20. 10.1371/journal.pbio.1000497 20957188 PMC2950128

[54] Ohno S. Gene duplication and the uniqueness of vertebrate genomes circa 1970–1999. Semin Cell Dev Biol. 1999;10(5):517–22. 10.1006/Scdb.1999.0332 PubMed PMID: ISI:000083733400010. 10597635

[55] Dusso A, Brown A, Slatopolsky E. Vitamin D. Am J Physiol-Renal. 2005;289(1):F8–F28. 10.1152/ajprenal.00336.2004 PubMed PMID: 000229741900002. 15951480

[56] Carlberg C. Ligand-mediated conformational changes of the VDR are required for gene transactivation. The Journal of steroid biochemistry and molecular biology. 2004;89-90(1–5):227–32. Epub 2004/07/01. 10.1016/j.jsbmb.2004.03.112 . 15225776

[57] Thompson PD, Remus LS, Hsieh JC, Jurutka PW, Whitfield GK, Galligan MA, et al Distinct retinoid X receptor activation function-2 residues mediate transactivation in homodimeric and vitamin D receptor heterodimeric contexts. Journal of molecular endocrinology. 2001;27(2):211–27. Epub 2001/09/21. . 11564604 10.1677/jme.0.0270211

[58] Peleg S, Sastry M, Collins ED, Bishop JE, Norman AW. Distinct conformational changes induced by 20-epi analogues of 1 alpha,25-dihydroxyvitamin D3 are associated with enhanced activation of the vitamin D receptor. The Journal of biological chemistry. 1995;270(18):10551–8. Epub 1995/05/05. . 7737990 10.1074/jbc.270.18.10551

[59] Bettoun D, Burris T, Houck K, Buck D, Stayrook K, Khalifa B, et al Retinoid X receptor is a nonsilent major contributor to vitamin D receptor-mediated transcriptional activation. Mol Endocrinol. 2003;17(11):2320–8. 10.1210/me.2003-0148 PubMed PMID: 000186273700016. 12893883

[60] Thompson PD, Jurutka PW, Haussler CA, Whitfield GK, Haussler MR. Heterodimeric DNA binding by the vitamin D receptor and retinoid X receptors is enhanced by 1,25-dihydroxyvitamin D3 and inhibited by 9-cis-retinoic acid. Evidence for allosteric receptor interactions. The Journal of biological chemistry. 1998;273(14):8483–91. Epub 1998/05/09. . 9525962 10.1074/jbc.273.14.8483

[61] MacDonald PN, Dowd DR, Nakajima S, Galligan MA, Reeder MC, Haussler CA, et al Retinoid X receptors stimulate and 9-cis retinoic acid inhibits 1,25-dihydroxyvitamin D3-activated expression of the rat osteocalcin gene. Molecular and cellular biology. 1993;13(9):5907–17. Epub 1993/09/01. 8395017 10.1128/mcb.13.9.5907-5917.1993 PMC360339

[62] Heery DM, Kalkhoven E, Hoare S, Parker MG. A signature motif in transcriptional co-activators mediates binding to nuclear receptors. Nature. 1997;387(6634):733–6. Epub 1997/06/12. 10.1038/42750 . 9192902

[63] Jurutka PW, Thompson PD, Whitfield GK, Eichhorst KR, Hall N, Dominguez CE, et al Molecular and functional comparison of 1,25-dihydroxyvitamin D(3) and the novel vitamin D receptor ligand, lithocholic acid, in activating transcription of cytochrome P450 3A4. J Cell Biochem. 2005;94(5):917–43. 10.1002/jcb.20359 . 15578590

[64] Bouillon R, Carmeliet G, Verlinden L, Van Etten E, Verstuyf A, Luderer HF, et al Vitamin D and Human Health: Lessons from Vitamin D Receptor Null Mice. Endocrine Reviews. 2008;29(6):726–76. 10.1210/er.2008-0004 18694980 PMC2583388

[65] Nagpal S, Na S, Rathnachalam R. Noncalcemic actions of vitamin D receptor ligands. Endocr Rev. 2005;26(5):662–87. 10.1210/er.2004-0002 PubMed PMID: 000230848500004. 15798098

