# Supplementary material for: Molecular Cloning, Functional Characterization, and Evolutionary Analysis of Vitamin D Receptors Isolated from Basal Vertebrates
Source: PLoS One. 2015 Apr 9;10(4):e0122853. doi: 10.1371/journal.pone.0122853 (PMC4391915; doi:10.1371/journal.pone.0122853)
Supplement: S1 Table — (PDF) [file pone.0122853.s001.pdf]

Table S1. List of primers.

Species	Vector	R.S.[1]	D[3]	Sequence
Lamprey VDR	pET32a	SalI	F	5' – GATAGTCGACTGATGATGGCCACTCAGACC – 3'
		NotI	R	5' – GATTGCGGCCGCTGCGGTTGGGTTGCCAAA – 3'
Skate VDR	Outer	N/A[2]	F	5' – GCAGGAAGCTGATTTCCAAG – 3'
		N/A	R	5' – CCAAGCCTGTTTACCCTGTG – 3'
	pSG5	BamHI	F	5' – AGCTAGGATCCATGGAACAGATGGCAGTG – 3'
		BglII	R	5' – ACGTAAGATCTTCATTTCGCATCATCATT – 3'
	pVP16	BamHI	F	5' – AGCTAGGATCCTTATGGAACAGATGGCAGT – 3
		HindIII	R	5' – ACGTAAAGCTTTCATTTCGCATCATCATT – 3'
	pET32a	SalI	F	5' – GATAGTCGACTGATGGAACAGATGGCAGTG – 3'
		NotI	R	5' – GATTGCGGCCGCTTTCGCATCATCATTACT – 3'
Bichir VDR	pSG5/ pVP16	EcoR1	F	5' – AGCTGAATTCATGGCAGCCATATCAGTG – 3'
		BamHI	R	5' – AGCTGGATCCCTAAGACACTTCATTGCC – 3'
	pET32a	SalI	F	5' – GATAGTCGACTGATGGCAGCCATATCAGTG – 3'
		NotI	R	5' – TATCGCGGCCGCAGACACTTCATTGCCAAA – 3'
Human VDR	pVP16	EcoRI	F	5' – GATAGAATTCATGGAGGCAATGGCGGCCAG – 3'
		BamHI	R	5' – TATCGGATCCTCAGGAGATCTCATTGCCAA – 3'
	pET32a	SalI	F	5' – CATAGTCGACTGATGGAGGCAATGGCGGCCAG – 3'
		NotI	R	5' – TATGCGGCCGCGGAGATCTCATTGCCAAACAC – 3'

[1]R.S. = restriction site
[2]N/A = not applicable.
[3]D = direction
