# Supplementary material for: Molecular Cloning, Functional Characterization, and Evolutionary Analysis of Vitamin D Receptors Isolated from Basal Vertebrates
Source: PLoS One. 2015 Apr 9;10(4):e0122853. doi: 10.1371/journal.pone.0122853 (PMC4391915; doi:10.1371/journal.pone.0122853)
Supplement: S2 Table — (PDF) [file pone.0122853.s002.pdf]

Table S2. GenBank Accession Numbers.

Name	Species	Accession #
Sea Lamprey VDR	*Petromyzon marinus*	AY249863
Elephant Shark VDR	*Callorhinchus milii*	XM_007910510.1
Little Skate VDR	*Leucoraja erinacea*	KJ925051
Frog VDR	*Xenopus laevis*	U91846
Alligator VDR	*Alligator mississippiensis*	XM_006273342.1
Chicken VDR	*Gallus gallus*	NM_205098
Rat VDR	*Rattus norvegicus*	J04147
Human VDR	*Homo sapien*	NM_000367.2
Senegal Bichir VDR	*Polypterus senegalus*	KJ925050
Spotted Gar VDR	*Leucoraja erinacea*	XM_006629244.1
Blind Cave Fish VDRα	*Astyanax mexicanus*	XM_007240812.1
Blind Cave Fish VDRβ	*Astyanax mexicanus*	XM_007249122.1
Zebrafish VDRα	*Danio rerio*	KJ925048
Zebrafish VDRβ	*Danio rerio*	KJ925049
Stickleback VDRα	*Gasterosteus aculeatus*	KM273008
Stickleback VDRβ	*Gasterosteus aculeatus*	KM273009
Flounder VDRα	*Paralichthys olivaceus*	AB037674.1
Flounder VDRβ	*Paralichthys olivaceus*	AB037673.1
Medaka VDRα	*Oryzias latipes*	EU403115
Medaka VDRβ	*Oryzias latipes*	EU403116
Pufferfish VDRα	*Takifugu ruripes*	XM_003962978.1
Pufferfish VDRβ	*Takifugu ruripes*	XM_003973646.1
Pufferfish VDRα	*Tetraodon nigroviridis*	KM273010
Pufferfish VDRβ	*Tetraodon nigroviridis*	KM273011
Carp VDR	*Cyprinus carpio*	AJ784084
Salmon VDR	*Salmo salar*	AJ780914
