# Supplementary material for: Molecular Cloning, Functional Characterization, and Evolutionary Analysis of Vitamin D Receptors Isolated from Basal Vertebrates
Source: PLoS One. 2015 Apr 9;10(4):e0122853. doi: 10.1371/journal.pone.0122853 (PMC4391915; doi:10.1371/journal.pone.0122853)

Supplementary File S3

Uncropped EMSA gel images for the canonical VDRE

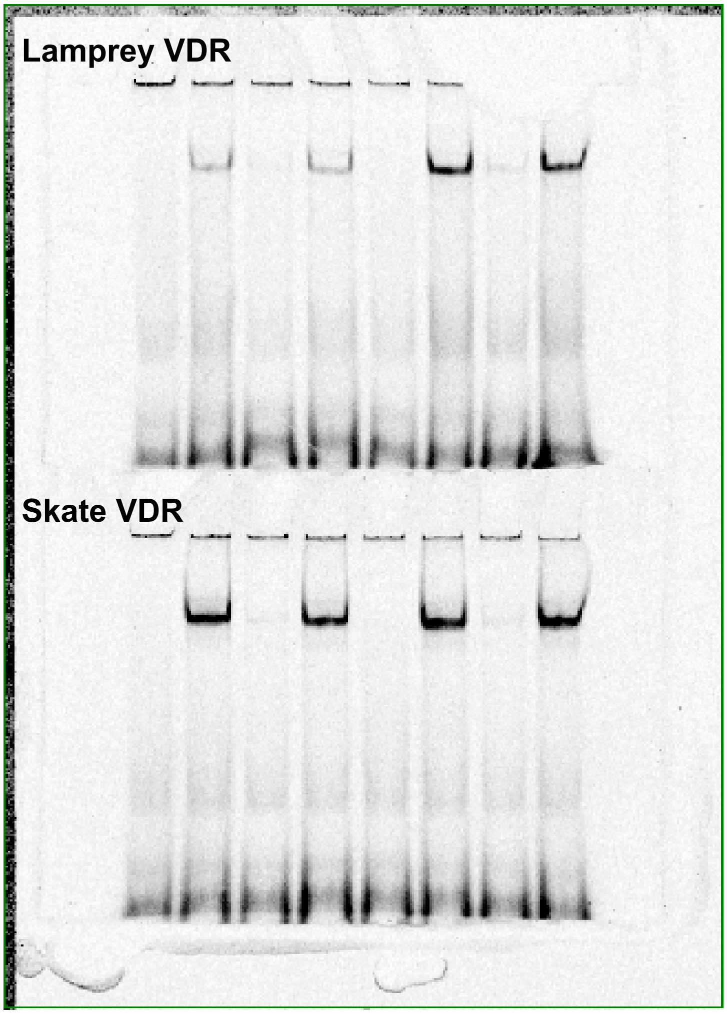

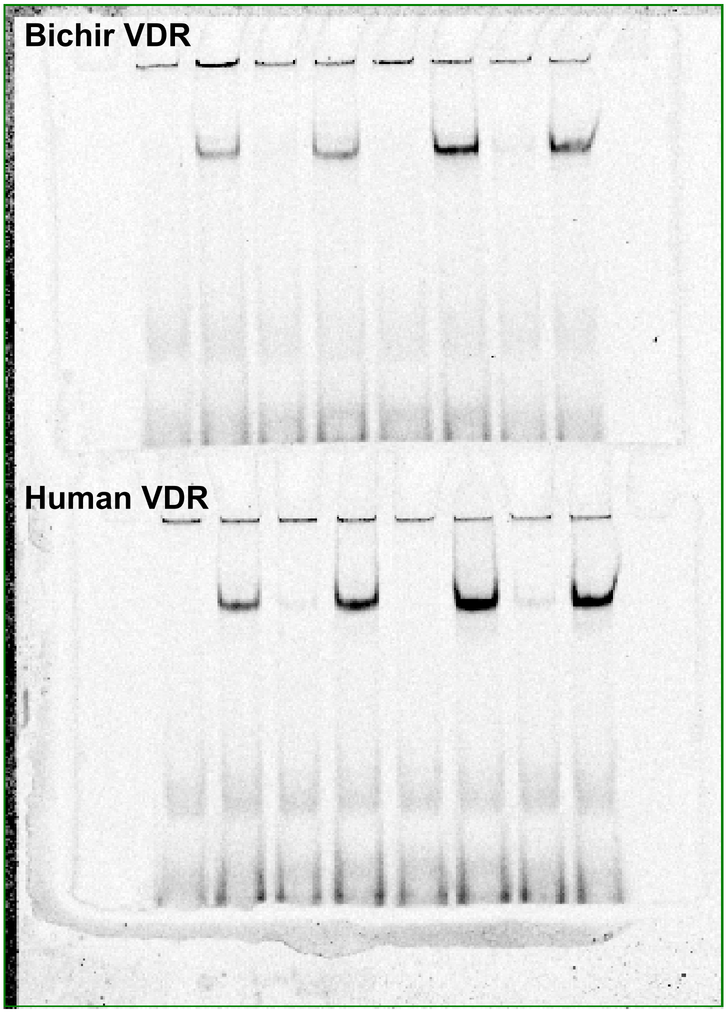

Supplementary File S3

Uncropped EMSA gel images for the XREM VDRE

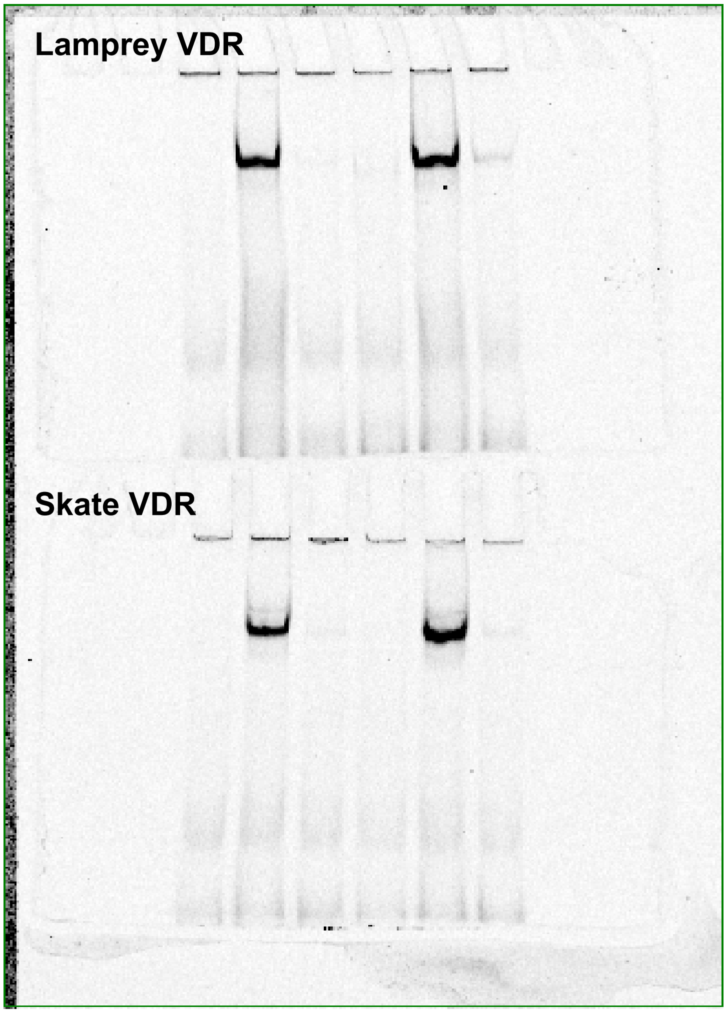

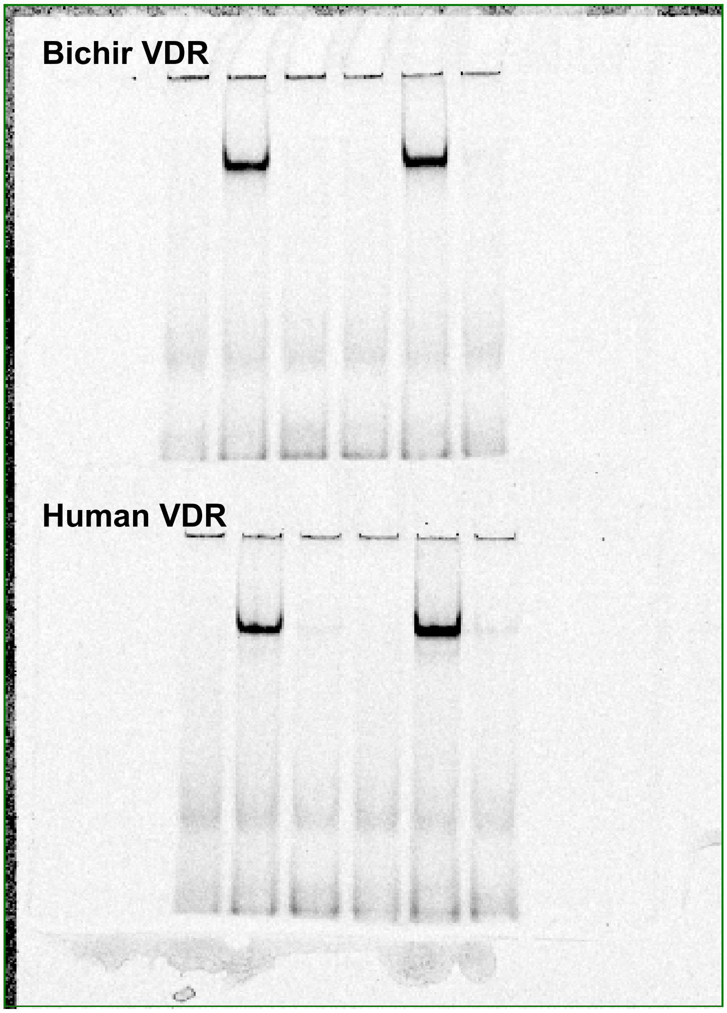

Supplement: S1 File — (PDF) [file pone.0122853.s003.pdf]
